# The epithelium takes the stage in asthma and inflammatory bowel diseases

**DOI:** 10.3389/fcell.2024.1258859

**Published:** 2024-03-11

**Authors:** Rocío López-Posadas, Dustin C. Bagley, Carlos Pardo-Pastor, Elena Ortiz-Zapater

**Affiliations:** ^1^ Department of Medicine 1, University Hospital of Erlangen, Friedrich-Alexander-Universität Erlangen-Nürnberg, Erlangen, Germany; ^2^ Deutsches Zentrum für Immuntherapie, Friedrich-Alexander-Universtiy Eralngen-Nürnberg, Erlangen, Germany; ^3^ Randall Centre for Cell and Molecular Biophysics, New Hunt’s House, School of Basic and Medical Sciences, Faculty of Life Sciences and Medicine, King’s College London, London, United Kingdom; ^4^ Department of Biochemistry and Molecular Biology, Universitat de Valencia, Valencia, Spain; ^5^ Instituto Investigación Hospital Clínico-INCLIVA, Valencia, Spain

**Keywords:** epithelium, barrier, mucus, asthma, IBD, therapeutics

## Abstract

The epithelium is a dynamic barrier and the damage to this epithelial layer governs a variety of complex mechanisms involving not only epithelial cells but all resident tissue constituents, including immune and stroma cells. Traditionally, diseases characterized by a damaged epithelium have been considered “immunological diseases,” and research efforts aimed at preventing and treating these diseases have primarily focused on immuno-centric therapeutic strategies, that often fail to halt or reverse the natural progression of the disease. In this review, we intend to focus on specific mechanisms driven by the epithelium that ensure barrier function. We will bring asthma and Inflammatory Bowel Diseases into the spotlight, as we believe that these two diseases serve as pertinent examples of epithelium derived pathologies. Finally, we will argue how targeting the epithelium is emerging as a novel therapeutic strategy that holds promise for addressing these chronic diseases.

## 1 Introduction

The epithelium has an essential role in development, physiology, and mucosal immunity. Its primary function is to act as a dynamic barrier, not only providing physical protection but also central to maintaining homeostasis and avoiding disease. Remarkably, despite experiencing high rates of cellular death and division, the epithelium maintains barrier function, underscoring the tissue’s need for precise spatial and temporal regulation. Healthy epithelial monolayers effectively shield against toxins, viruses, pollutants, pathogens, and a long list of insults and attacks. Notably, when the integrity of the monolayer is compromised, a range of disorders follow, many of which remain classified as inflammatory disease, such as asthma and Inflammatory Bowel Disease (IBD) that we discuss herein.

Epithelial barrier damage triggers manifold and complex, inter-connected mechanisms involving not only epithelial cells, but also other resident cells within the mucosa, including immune and stroma cells. Traditionally, the immune cell population has been viewed as the “police” of the barrier, and many diseases known to have damaged epithelium and dysfunctional barriers have long been regarded by the scientific community as “immunological diseases”. Subsequently, studies aimed at understanding, preventing, and treating these diseases have heavily relied on immune-centric therapeutic strategies that even though, effective at symptom management, cannot stop, nor revert, the disease’s natural progression. For example, targeting inflammation in asthma has been successful in managing major symptoms resulting in decreased exacerbation, hospitalization, and mortality (Rupani et al., 2021). However, it has been clearly demonstrated that these treatments do not impede the relentless progression of the disease, suggesting we are missing an underlying aetiology. Indeed, epithelial damage is seen in every type of asthma and is correlated with disease severity (Holgate, 2007; Lambrecht and Hammad, 2012; Calven et al., 2020; Porsbjerg et al., 2023). We can observe a similar situation in IBD, where past clinical practice has been restricted to symptom control using unspecific immunosuppressive drugs. But in the last years, the concept of mucosal healing has revolutionized the medical management of IBD patients, which goes beyond the symptom control towards the resolution of inflammation and ultimately complete healing (Rath et al., 2021; Neurath and Vieth, 2023). Thus, endoscopic and histological remission are nowadays considered as key therapeutic goals and prognostic parameters. More recent studies also argue for the importance of intestinal barrier healing in this context (Rath et al., 2023), highlighting again the role of epithelial function in the disease pathogenesis. In fact, several observations in the last 20–30 years support the causative role of epithelial alterations in IBD pathogenesis. For instance, there is a familial background in the increased intestinal permeability in IBD patients and their relatives (Munkholm et al., 1994; Soderholm et al., 1999; Irvine and Marshall, 2000), and the occurrence of epithelial leakage has been shown to be reliable for the prediction of IBD flares (Kiesslich et al., 2012), while does not correlate to inflammation severity (Benjamin et al., 2008). The lack of response to current therapy in chronic diseases, such as asthma or IBD, and the low safety profile of immunosuppressive drugs implies the need of alternative therapies. In fact, strategies targeting epithelial restoration emerge as attractive candidates and deserve further investigations.

In this review, we aim to discuss specific epithelial-driven mechanisms that ensure barrier function. These include 1) mechanisms related to the architecture and structure of the epithelium that regulate epithelial paracellular permeability, 2) the existence of a mucus layer that is able to eliminate particles and impact on the microbiota and 3) secretion of chemo/cytokines or antimicrobial substances (Ganesan et al., 2013; Mookherjee et al., 2020). We will discuss in detail the epithelium in the lungs and in the gut with the goal of understanding the different mechanisms named above and how those are dysregulated in respiratory and intestinal diseases, putting both asthma and IBD in the focus. We will also argue how targeting the epithelium is emerging as a new therapeutic strategy that could provide solution for these two chronic diseases and others.

## 2 Structure of the epithelial layer in the lung and in the gut

### 2.1 Cell types in the lung and gut epithelium

Epithelia are formed by a continuous layer of interconnected cells encapsulating organs and lining cavities. Epithelial cells are anchored to the *basal lamina* or basement membrane, a thin layer of extracellular matrix that provides structural support and signalling cues and sits on top of the underlying stromal tissue, which provides nutritional support and contains nerve terminals and immune cells that exchange signals with the epithelial sheet, capable of actively orchestrating and maintaining adaptive responses in health and disease (Lambrecht and Hammad, 2012).

**FIGURE 1:**
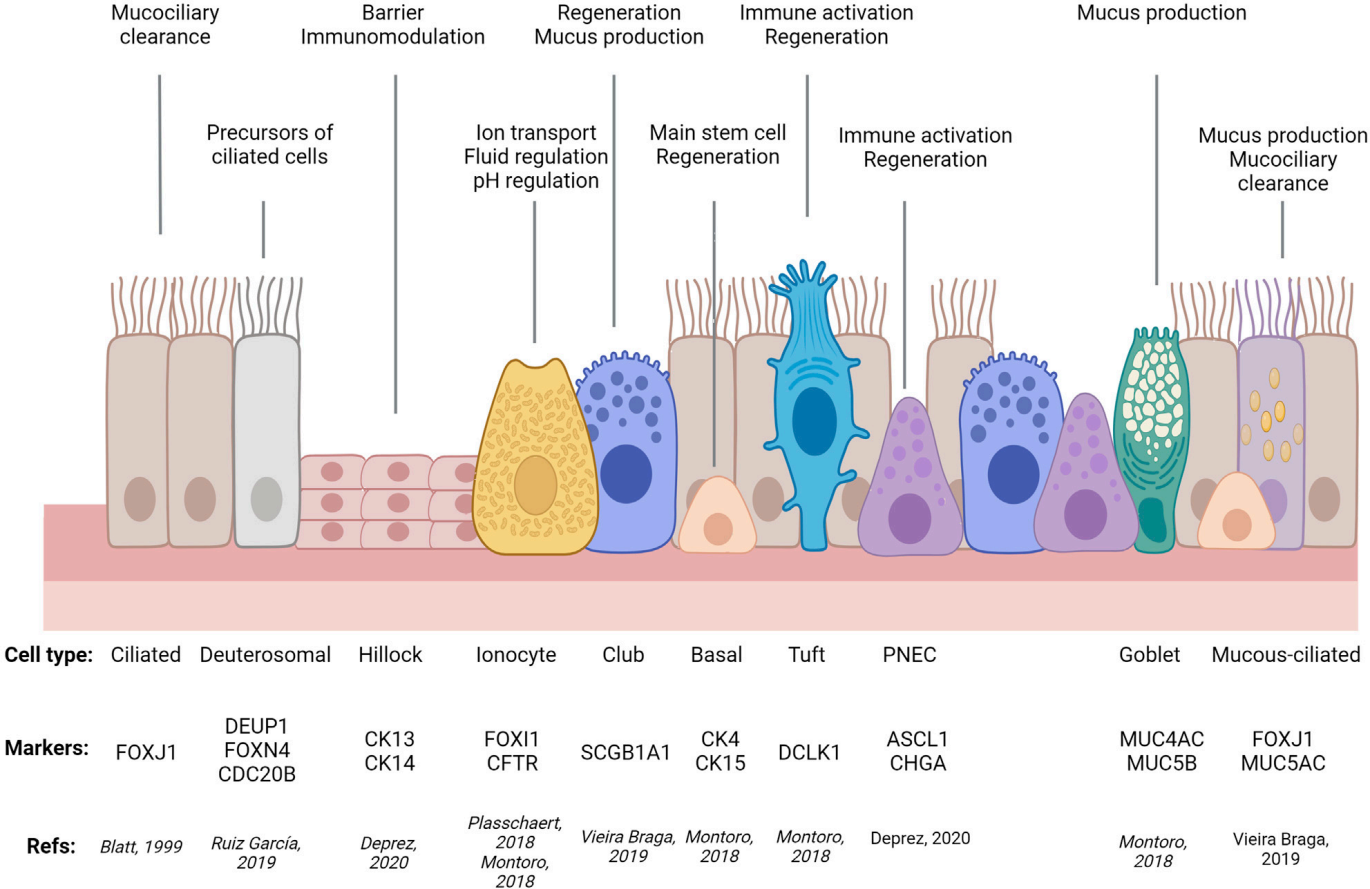
Simplified diagram of airway epithelial cell types, their molecular markers, and main functions. PNEC: Pulmonary Neuro-Endocrine Cell. Adapted from (Ortiz-Zapater et al., 2022a) using Biorender.com.

For decades, researchers relied on microscopy-based morphological criteria to define different epithelial cell types that, combined with tissue architecture, determine the balance between different epithelial functions: protective, absorptive, and/or secretory. As an example, airway ciliated cells were first described in 1837, followed in 1852 by description of cells lacking cilia, loaded with granules, with a narrow stem connected to the basement membrane by a circular structure (goblet cells) and two cell types lacking access to the airway lumen: spherical cells, adjacent to the basement membrane (basal cells) and two layers of elongated cells (intermediate cells) (Figure 1). Remarkably, these early studies already were able to appreciate cell type similitudes between different tissues and proposed basal cells were precursors of the other airway epithelial cell types (for comprehensive historical perspective of airway cell type discoveries, see (Widdicombe, 2019).

**FIGURE 2:**
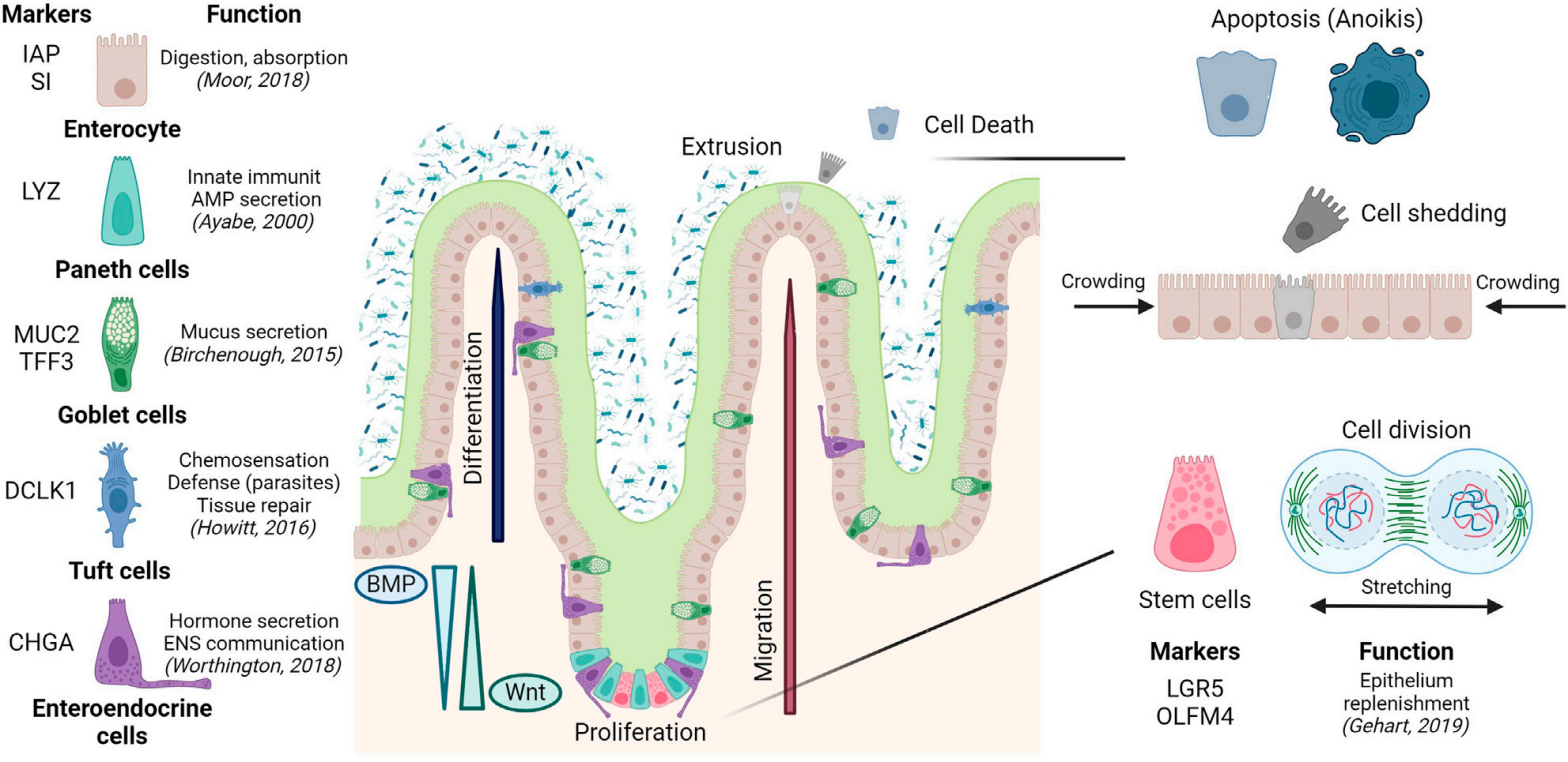
Epithelial composition and architecture in the small intestine, including epithelial turnover along the crypt-villus axis. Created with Biorender.com.

Later, development of molecular markers and transgenics offered functional criteria to further define these cell types, genealogies, and functions, and how all these depend on tissue architecture. The gut is an example with clear spatial segregation of division, differentiation, tissue-specific functions, and death. Intestinal stem cells residing at the bottom of the crypts give rise to transient-amplifying cells (Duckworth, 2021). These are pluripotent cells that sequentially differentiate into absorptive (enterocytes) and secretory lineages. The latter gives rise to different cell subtypes achieving pleiotropic functions: i) antimicrobial peptide-producing paneth cells, not present in the colon; ii) mucus secreting goblet cells; iii) enteroendocrine cells releasing hormones, and chemosensory tuft cells (Fre et al., 2005). Epithelial cell differentiation is linked to migration upwards from the crypt to the villus or surface epithelium; except for paneth cells in the small intestine, which remain at the crypt bottom in close connection with stem cells (Garabedian et al., 1997). Cell migration and compartmentalization of crypts and villus is regulated by the activation and/or gradient between different pathways (Wnt, EGF, Notch or BMP), in most cases due to the contribution of pericryptal cells and the sub-epithelial microenvironment (Reynolds et al., 2014; Chen et al., 2019). Finally, differentiated cells at the villus tip will be extruded into the lumen where they finally die, to allow the renewal of the epithelial layer or epithelial turnover (Watson et al., 2009) (Figure 2).

In recent years, single cell and spatial transcriptomics have redefined and expanded cell types in virtually all tissues analysed, highlighting commonalities and tissue-specific features, echoing Waymouth Reid’s conclusion that “it is extremely probable that several varieties of such [secreting] structures exist” and greatly contributing to the description of cellular complexity of the intestinal and respiratory epithelia. Single-cell RNA sequencing (sc-RNA-Seq) has confirmed the suggested variability in terms of cell composition and heterogeneity between organs and regions, e.g., small intestine vs. colon, crypt vs. villi, different airway regions, like trachea, airways or alveoli (McKinley et al., 2017; Beumer et al., 2018; Montoro et al., 2018; Moor et al., 2018; Plasschaert et al., 2018; Burclaff et al., 2022).

Additionally, sc-RNA-Seq has identified previously uncharacterised rare types of tissue-specific cells (e.g., lung ionocytes (Montoro et al., 2018; Plasschaert et al., 2018) and ones shared by different epithelia (e.g., tuft cells in airways, gastrointestinal tract, and other tissues (Elmentaite et al., 2021). Importantly, these techniques have demonstrated that the transcriptional profile between different cell subtypes, and thus our distinction between secretory and absorptive (gut) or ciliated types (airways), is not as clear as previously thought. Examples of this are colonic deep secretory cells contributing to the stem cell niche but with classical markers of differentiated goblet cells (Parikh et al., 2019) or mucous-ciliated and suprabasal cells in the airways (Elmentaite et al., 2021). Moreover, these techniques enable tracking of cells transitioning between states (trajectories), identifying new regulatory roles for Sox4, Foxm1, Mxd3, Batf2 in enterocytes (Haber et al., 2017) or Foxi1 in airway ionocytes (Montoro et al., 2018); as well as segregated populations within a given trajectory, such as tuft-2 cells displaying immunological functions (Haber et al., 2017). By enabling comparison between airway states (development, homeostasis, disease) these techniques have shed light on disease mechanisms like a general upregulation of secretory gene expression in all asthmatic airway epithelial types, in addition to a novel intermediate mucous-ciliated cell state expressing markers of both classic cell types that, with goblet cell hyperplasia, contributes to mucous hyperplasia in asthma. In the gut, the same approach has also identified defective mucus maturation in goblet cell as a potential driver of IBD and colorectal cancer, in addition to a new pH-sensing absorptive cell type, pericryptal stromal signalling, lymphocyte imbalance, and platelet aggregation as key contributors to barrier dysfunction in IBD (Regev et al., 2017; Beumer et al., 2018; Kinchen et al., 2018; Huang et al., 2019a; Parikh et al., 2019; Vieira Braga et al., 2019; Deprez et al., 2020; Jackson et al., 2020; Travaglini et al., 2020; Beumer and Clevers, 2021; Elmentaite et al., 2021; Haniffa et al., 2021; Tang et al., 2022).

All this demonstrates how recent advances in genomics, cell lineage tracing, and sc-RNA-Seq have revealed not only the need to redefine the meaning of cell identity, but also have uncovered new cell types involved in epithelial homeostasis and disease.

### 2.2 No cell is an island: how to build a monolayer from a single cell

Cell-cell junctions weave single epithelial cells into a functioning and dynamic monolayer that acts as a polarized barrier while selectively allowing transepithelial movement of water, ions, and macromolecules. Physiological transepithelial transport is classified as transcellular (mediated by transporters in apical and basolateral membranes) and paracellular transport (mainly determined by tight junctions). In the later, pore and leak pathways act in an interdependent manner (Weber et al., 2010). All these aspects have been nicely reviewed recently (Horowitz et al., 2023). Conversely, in damaged epithelia, transport becomes unrestricted and unselective, even allowing passage of bacteria from the lumen to the underlying tissue.

According to their location, composition, and function, epithelial intercellular junctions are classified as tight junctions (TJs), adherens junctions (AJs) or desmosomes, but all have common features like transmembrane components that physically link neighbour cells, in complex with cytoplasmic scaffolding and adaptor proteins linking the junctions to the cytoskeleton, which confers them mechanosensitivity (Garcia et al., 2018; Beutel et al., 2019; Pannekoek et al., 2019; Angulo-Urarte et al., 2020; Haas et al., 2022). TJs are formed by homotypic claudin and occludin contacts at the apex of lateral membranes between contacting cells. TJs form a regulable belt around a cell, separating the apical and basolateral membrane domains, while also sealing the paracellular pathway to control water and solute diffusion. The cytoplasmic side of TJs binds to adaptor proteins (ZO-1, -2, -3, cingulin) that interact with microtubules and the cytoskeleton. AJs are formed by the calcium-dependent extracellular trans binding of cadherins and force-dependent cytoplasmic binding to actin and microtubules via catenins. AJs are essential for cell-cell adhesion and epithelial mechanical responses, detailed later. Desmosomes are strong intercellular junctions based on cadherins desmoglein and desmocolin, bound to intermediate filaments via catenins plakoglobin and plakophilin. Moreover, junctions act also as signalling hubs, in close interconnection with Rho GTPases (Citi et al., 2014). Small GTPases are frequently found inactive, bound to GDP. After GDP-GTP replacement by Guanine Exchange Factors (GEFs), GTPases are recruited and interact with effector proteins, regulating essential cell functions controlling cell-cell adhesion and barrier function like mechanotransduction, vesicle trafficking, or junctional component dynamics (Braga, 2018). In summary, junctional integrity is essential for epithelial function, and its disruption is a key aspect of diseases like asthma and IBD.

In fact, mechanotransduction between epithelial cells determines tissue homeostasis at different levels. External forces (breathing, circulation flow, peristaltic movements), GTPases, and cytoskeletal contractility control long-term biological outcomes at cell (identity, proliferation, migration, extrusion) and tissue levels (folding, compartmentalization) in development, differentiation, homeostasis, and repair at the cell (identity, proliferation, migration and extrusion) and tissue levels (folding, compartmentalization) (Mahoney et al., 2014; Zhao et al., 2014; Goodwin and Nelson, 2021; Alvarez and Smutny, 2022; Perez-Gonzalez et al., 2022; He et al., 2023). Architecture of the gut epithelium represents a good example in this context; thus, myosin contractility initiates crypt invagination, the GTPase Rac1 controls crypt-villus compartmentalisation, and mechanical tension drives homeostatic intestinal cell migration from crypts to villus (Sumigray et al., 2018; Yui et al., 2018; Krndija et al., 2019; Perez-Gonzalez et al., 2021; Yang et al., 2021; Perez-Gonzalez et al., 2022).

As mentioned, mechanical forces also regulate cell identities, frequently via the transcriptional regulator YAP and its interplay with other signalling pathways, with remarkable tissue-specific features. In the gut, stiffening decreases stemness and promotes YAP-dependent gut stem cell differentiation into goblet cells (He et al., 2023); whereas in the lung, YAP is essential to maintain tissue organization and prevent stem cell loss and excessive goblet cell differentiation and mucin hypersecretion during homeostasis (Mahoney et al., 2014; Zhao et al., 2014; Hicks-Berthet et al., 2021). These differences could be partly explained by YAP being essential in all regenerative scenarios and lung homeostasis, but not in gut homeostasis (Camargo et al., 2007; Barry et al., 2013; Zhao et al., 2014; Yui et al., 2018; Hicks-Berthet et al., 2021).

Mechanical forces also regulate cell numbers in shorter time scales. Cell stretching signals through E-cadherin and Piezo1 to increase nuclear levels of YAP and β-catenin and CDK1 activity, driving cell cycle re-entry (Streichan et al., 2014; Benham-Pyle et al., 2015; Gudipaty et al., 2017; Uroz et al., 2018). Conversely, crowding or compression arrests cell cycle and restores homeostatic cell numbers via cell extrusion, an evolutionarily conserved mechanism where a supracellular actomyosin cable formed around the unwanted cell ratchets in and down, resulting in seamless cell eviction without compromising barrier function (Rosenblatt et al., 2001; Eisenhoffer et al., 2012; McClatchey and Yap, 2012; Puliafito et al., 2012). In that sense, extrusion also works as an innate defence mechanism against external aggression, with healthy cells collectively squeezing cells infected by bacteria or viruses, thus limiting pathogen spreading in the monolayer and ensuring epithelial barrier function (Bastounis et al., 2021; Hippee et al., 2021; Lin et al., 2021; Moshiri et al., 2023).

## 3 The mucus and the secretome: let’s keep it wet and clean!

Epithelial barrier function is not limited to a single sheet of interconnected epithelial cells; a layer of mucus coats the apical side of these cells and acts as a first barrier coating internal surfaces of organs. In turn, epithelial cells not only act to form a barrier. Instead, they communicate with other cell types, including immune or stromal cells, via secreted molecules, what can be defined as the “epithelium secretome”. For a detailed description of the evolution of the cell secretome, we recommend (Sanchez-Guzman et al., 2021).

### 3.1 The mucus

Both the gut and the respiratory epithelium luminal surface are protected by mucus, a selective barrier to particles and molecules that is built around a family of polymeric glycoproteins called mucins. Mucus that coats the epithelium is a complex hydrogel biopolymer barrier, present not only in the airways and the gastrointestinal tract, but also in the reproductive tract and eyes (Lieleg and Ribbeck, 2011). During homeostasis, the protective mucus layer is produced by the goblet cells that are equipped with specific biological machinery for the secretion of mucins. Notably, some respiratory diseases are characterised by changes in goblet cells function (like asthma or COPD, see Table 1) and we will discuss later the importance of mucus production dysregulation in the pathology of asthma and IBD.

**TABLE 1:** Examples of mucus-related diseases and alterations.

Alteration in which mucus-related components?	Disease	References
GUT (Main mucin: MUC2)		
Alteration of the mucin O-glyocosylation profile	IBD and colorectal cancer	Etienne-Mesmin et al. (2019)
Increase of mucin degradating bacteria such as Ruminococcus family	Ulcerative colitis	Hansson, (2019)
loss of mucus viscoelastic properties and consequently a loss of protective function	Crohn’s disease	Cornick et al. (2015)
LUNG (Main mucins: MUC5B and MUC5AC)		
Alterations in the CTFR channel in the Goblet cells, mucin hyperconcentration and corresponding impaired mucus clearance	Cystic fibrosis	Gustafsson et al. (2012), Henderson et al. (2014), Hill et al. (2018)
Trapped mucus in the epithelium	Asthma, COPD	Fahy et al. (1993)
Increased ratio of MUC5AC to MU5B	Pediatric asthma	Welsh et al. (2017)
Elevated sputum production with both MUC5AC and MUC5B	Non-CF bronchiectasis	Ramsey et al. (2020)
Reduced MUC5B	Pulmonary alveolar proteinosis	Takeyama K et al. (2015)

In the gut, mucus offers moisturising and lubricant properties, protecting the epithelial cells from dehydration and mechanical stress during the passage of luminal content and peristalsis forces (Johansson et al., 2013). It also operates as a surface cleaner, removing debris and bacteria, through binding, collecting, and flushing them away via intestinal flow. The small intestine has a single layer of mucus; while in the stomach and colon, the mucus layer is composed by an inner layer, attached to the epithelium, and an outer layer that interacts with luminal components. The inner layer is impermeable to bacteria and renewed by globlet cells every hour. The outer mucus layer is less dense and is the habitat for commensal bacterial (Hansson, 2019). Notably, in the small intestine, mucus leaves pores that allow the bacteria to penetrate, which is not the case in the large intestine, where the mucus layer is thick and completely avoids the contact with bacteria and the epithelial cells (Paone and Cani, 2020). In the gut, the main mucin is MUC2, which composes the skeleton of the mucus layer. In addition to MUC2, the IgG Fc-binding protein, FCGBP and the intestinal trefoil factor, TFF3 act synergistically to enhance the mucus barrier and exert antibacterial effects, while the metalloenzyme CLCA1 is involved mainly in the stratification and expansion of mucus. Moreover, ZG16, RELMβ, Lypd8, sIgA, and AMP exert bacteriostatic or bactericidal effects under different conditions (Song et al., 2023).

In the airway, mucus is composed of water, different proportions of polymerizing mucin glycoproteins MUC5B and MUC5AC in proximal *versus* distal regions (Meldrum and Chotirmall, 2021), a range of antimicrobial molecules (defensins, lysozyme, etc.), cellular debris including DNA, and protective factors (trefoil factors) (Thornton et al., 2008). The protective response is driven by microbial sensors in the goblet cells that initiate secretion of mucus, to entrap invading microbes and remove bacteria away through mucociliary clearance (Abdullah et al., 2018). The ciliated cells, which line the surface epithelium of the airways, provide the force necessary for mucociliary clearance by the coordinated beating of their cilia, which confers an escalator motion to bring unwanted material to the mouth to be coughed out. These highly specialized cells are therefore critical to the health and function of the pulmonary system, and often preferential destroyed in favour of mucus producing cells in pathologies like asthma, with mucus hyper-production and -secretion remaining a massive obstacle in asthma treatment.

Many diseases arise from an imbalance between mucus production and elimination. The role of mucus and mucins in diseases of the intestinal and respiratory tracts is excellently reviewed by Hansson and others (Hansson, 2019) and we will describe later the specific importance of mucus regulation in asthma and IBD. We have included in Table 1 other diseases showing specific alteration linked to respiratory or intestinal diseases and the link to different respiratory and digestive diseases. See also (Meldrum and Chotirmall, 2021) for additional information.

It is now clear that the maturation and function of the mucus layer are strongly influenced by the microbiota (Schroeder, 2019). In fact, the consideration of the microbiota as a continuous element of homeostatic regulation of the epithelium has undoubtedly made physicians and researchers to confirm the relationship between the microbes and the epithelial barrier, and to adopt a more holistic view of the disease (Runge and Rosshart, 2021).

On of the main factors that influences the presence of a specific microbiota is the composition of the mucosal layer. Indeed, the mucin glycosylation profile influences the composition of mucus-associated bacteria, selecting specific species (Bergstrom and Xia, 2013). The composition of the mucus not only controls bacteria adhesion, but mucin glycans can also serve as nutrients for specific microorganisms, depending on their glycan-degrading enzyme’s content, highlighting an example of how the host controls the microbiota within the mucus layer (Paone and Cani, 2020). Finally, bacteria can use host glycans to form new polymers used in the creation of their capsule, promoting evasion from the immune system (Martens et al., 2009).

Factors like age, diet, drugs, or disease affect microbiota composition too, even compromising its barrier function. For example, during pulmonary infection, microbial dysbiosis leads to invasion by opportunistic pathogens. These communities disrupt tissue compartments within the airway lumen, including mucus and causing progressive, localized, and chronic infection, particularly in pulmonary diseases (Montassier et al., 2023). Moreover, in asthma, exacerbations are classically induced by infections, like the ones produced by *P.* aeruginosa, which disrupts pulmonary mucins significantly contributing to disease progression (Meldrum and Chotirmall, 2021). Although the association between IBD and dysbiosis is accepted, whether alterations of the microbiota represent a cause or consequence of the disease is still a matter of discussion (Palm et al., 2014; Forbes et al., 2016; Schaubeck et al., 2016; Becker et al. 2015).

### 3.2 The secretome: secreted molecules in the gut and lung

**FIGURE 3:**
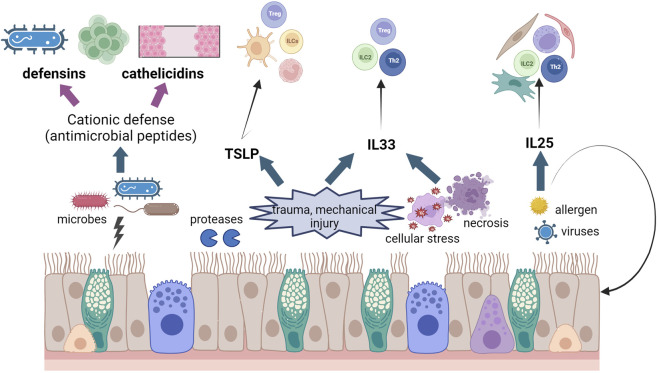
Schematic diagram of effects of epithelial damage in asthma and IBD. We have illustrated the release of CHDP and cytokines from the epithelium after different insults and the different cell populations that these molecules activate. Created using Biorender.com.

Epithelial cells produce and secrete several molecules that contribute to epithelial integrity and elimination of microorganisms and contaminants, as well as intercellular communication. These molecules, collectively known as “the secretome” support epithelial homeostasis by controlling important cellular processes like proliferation, different mechanisms of cell death, safeguarding of epithelial tight junctions, maintenance of a healthy microbiota, and of course, communication with other cell types, like immune or stromal cells. We will cover some of the key players in the epithelium secretome in the gut and lung and we have summarised their main function in Figure 3.

#### 3.2.1 Cationic host defense peptides (CHDP)

CHDP are one of the major components of the inmate immunity both in the lungs and in the gut. Also known as antimicrobial peptides, CHDP are amphipathic peptides that combat infections through their direct microbicidal properties and/or by influencing the host’s immune responses. There are two main classes of CHDP in vertebrates, defensins and cathelicidins, produced as prepropeptides later cleaved to yield mature active peptides (Mookherjee et al., 2020). The last 25 years have seen an increasing interest in using CHDP as therapeutical targets, with potential clinical uses for asthma (Piyadasa et al., 2018) or colitis (Ho et al., 2013) treatment.

Defensins are key effector molecules in host defense against infection due to their broad-spectrum, and they contribute specially to the defense in the skin, lung, and gut. Defensins form producing destructive pores in the membrane of pathogens, and are also involved in inflammation, modulation of immune responses, wound repair, and disease (Weber, 2014). Epithelial cells are the main cellular sources, but they are also produced by neutrophils and other immune cells (Hiemstra, 2006). The main defensins produced by the epithelial cells in the respiratory tract and the gut are the β-defensins, with human β-defensin 2 mutations associated to asthma and atopy in children (Borchers et al., 2021) and their inhibition suppressing features of asthma in murine models (Pinkerton et al., 2021). Defensins produced by paneth cells in the small intestine contribute to tissue homeostasis by directly affecting the microbiota composition, but also by regulating the function of immune cells. In fact, reduced α- and increased β-defensins expression, as well as imbalance between the different mocules in therm of expression have been detected in the gut of IBD patients (Wehkamp et al., 2005; Elphick et al., 2008; Simms et al., 2008). In addition, a gene cluster polymorphism with low gene copy number of β-defensin-2 shows a predisposition for colonic CD (Fellermann et al., 2006).

Cathelicidins are also produced by epithelial cells of the respiratory and gastrointestinal tracts, but also by keratinocytes and neutrophils (Mookherjee et al., 2020). Cathelicidins have been studied in asthma in relation with viral-induced exacerbations, as its level could be used as a predictor marker (Arikoglu et al., 2017). In the gut, the cathelicidin LL37 has been shown to have a protective role and it has been postulated as a biomarker of pediatric IBD (Krawiec and Pac-Kozuchowska, 2021).

#### 3.2.2 Cytokines

TSLP (thymic stromal lymphopoietin), interleukin 33 (IL33) and interleukin 25 (IL25) are three typical epithelial cytokines that contribute to epithelial homeostasis and alert the immune system to external insults in order to regulate tissue restoration and repair (Ham et al., 2022; Mahapatro et al. 2021; Roan et al. 2019). These three “alarmin” cytokines are specifically potent in activating type 2 innate lymphoid cells (ILC2s) and therefore their roles have been widely studied in allergic inflammation and exacerbations, as well as parasite infections in the gut (Hammad and Lambrecht, 2015; Topczewska et al., 2023). Amplification or intensification of their secretion signals lead to different inflammatory diseases that we have tried to summarise in Table 2.

**TABLE 2:** Summary of characteristic of main epithelial cytokines.

Alarmin	Produced by	Produced because of…	Main targets	Related disease in the respiratory or gut epithelium	Receptor and signaling pathway
TSLP	Epithelial, stromal, dentritic cells, mast cells and basophils	Infection, inflammation, trauma, mechanical injury or proteases such as trypsin and papain (Allakhverdi et al., 2007)	Dendritic cells, Tregs, basophils and innate lymphoids cells (ILCs) (Roan et al., 2019)	Asthma, allergic rhinoconjunctivitis, nasal polyposis, COPD, esophagitis, gastrointestinal allergy, ulcerative colitis and Chron’s Disease	Heterodimer receptor, TSLPR/IL-7Ra, recruitment of JAK1 and JAK2 and activation of STAT5 that is translocated to the nucleus
IL33	Epithelial, endothelial, smooth muscle cells, fibroblasts, platelets and mast cells	Cellular stress, injury or necrosis	ILC2s, memory Th2 cells and Tregs (Salimi et al., 2013; Halim et al., 2014; Vasanthakumar et al., 2015)	Asthma, COPD, gastrointestinal allergy, ulcerative colitis, Chron’s Disease	Heterodimer receptor, formed by ST2 and IL-1RAP and activation of MYD88. This can activate both the NF-kB or the AP-1 pathway
IL25	Lung epithelial cells, endothelial cells, fibroblasts, alveolar macrophages, mast cells, basophils, eosinophils, chemosensory cells in the nasal mucosa	Allergen and viruses	T cells, ILC2s, Natural Killer Cells (NK), fibroblasts, epithelial, mesenchymal or endothelial cells (Stock et al., 2009; Saenz et al., 2010; Huang et al., 2015; Roan et al., 2019)	Asthma, atopic disease	Heterodimer receptor composed of IL17RA and IL17 RB. Binding recruits the adaptor proteins, such as ACT1 and TRAF6, and then activates NF-kB, MAPK-ERK and JNK.

TLSP is a member of the IL2 cytokine family mainly produced by epithelial cells in the lungs, but also by other cells types like intestinal tuft-2 cells, an example of finding made possible by sc-RNASeq techniques (see Table 2 and (Kashyap et al., 2011; Roan et al., 2019). Basal TSLP secretion is increased by several stimuli, although the existence of two isoforms of TSLP, long and short, may indicate status-dependent expression and secretion in homeostasis and disease. This has been studied in mice but its conservation in humans and the functional consequences of the variants remain unknown (Fornasa et al., 2015). Several publications have shown that a TSLP/ILC axis may play a pivotal role in steroid-resistant allergic airway inflammation (Kabata et al., 2013; Liu et al., 2018), very important in the treatment of asthma. IL33 is enriched in the barrier surfaces of the skin, lung, and intestine. Epithelial, and endothelial, cells express IL33 constitutively in the nucleus. Although some studies suggest that IL33 could have a role as a transcription factor (Ali et al., 2011), the nuclear localization is better explained as a mechanism to fine the release of this cytokine (Travers et al., 2018). IL33 can be present as a full-length protein, but proteolytic cleavage by other cell types or by molecules as caspases can produce both its activation or inactivation (Luthi et al., 2009; Lefrancais et al., 2014; Clancy et al., 2018). TSLP and IL33 have been suggested as protective molecules in IBD (Taylor et al., 2009). UC patients show reduced expression of TSLP (Tahaghoghi-Hajghorbani et al., 2019) and controversial data are available concerning IL33 in CD and UC (Seidelin et al., 2010; Tahaghoghi-Hajghorbani et al., 2019). Finally, IL25 can be secreted by specific subtypes of epithelial cells (Kohanski et al., 2018), but also other cell types, such as mastocytes and macrophages (Ikeda et al., 2003; Kang et al., 2005). In the gut, tuft cells are the main source of this cytokine (von Moltke et al., 2016). IL25 is secreted as a disulfide-linked homodimer. The activity of IL25 can be regulated by the matrix metalloproteinase, MMP7, which can cleave IL25 (Goswami et al., 2009); and also by splicing mechanisms. Although these three cytokines share target cells and have been implied in promoting type 2 inflammation, it could be interesting to understand what the interplay is among the three of them is, and whether pattern of expression of these epithelia cell-derived cytokines may distinguish distinct allergic endotypes or phenotypes.

There are other cytokines produced by the barrier epithelium that we cannot cover in this review. For example, cigarette smoke, another important insult for the barrier, and also other inhaled irritants promote expression and release of inflammatory mediators such as tumor necrosis factor (TNFα), IL1β, CXCL8 or the granulocyte-macrophage colony-stimulating factor, GM-CSF (Gao et al., 2015). The attenuation of GM-CSF signalling has been seen to decrease allergic inflammation in different mice models (Sheih et al., 2017). IL18 has been shown to be critical in driving the pathological breakdown of barrier integrity (Nowarski et al., 2015). On the other hand, IL-1α, produced by keratinocytes, can drive chronic skin inflammation (Archer et al., 2019).

#### 3.2.3 TGFβ

Finally, another important molecule for the communication between epithelial cells and stromal cells in the context of the extracellular matrix (ECM) remodelling that can occur after dysfunction of the epithelial barrier is the transforming growth factor β (TGFβ). The role of TGFβ has been extensively studied in the epithelium, where it enhances epithelial barrier dysfunction, cell differentiation or epithelial to mesenchymal transition (Kahata et al., 2018). TGFβ is secreted in an inactive form bound to the latency-associated peptide (LAP) and its activation requires conformational changes leading to the protein cleavage of LAP (Bauche and Marie, 2017). In its canonical pathway, TGFβ, in a dimeric form, binds to a tetrameric complex composed of TGFβ receptor I and II. The activated receptor phosphorylates Smad2/3 transcription factors, triggering their translocation to the nucleus (Meng et al., 2016) to regulate the transcription of several genes like collagens (I and IV) or fibronectin, components of the ECM (Huang et al., 2020). We believe that it is important to highlight the role of TGFβ as the main character of fibrosis, understanding fibrosis as an excessive way of healing a wound after putting at risk the epithelial barrier. This has been for example, demonstrated in mice models of asthma where disruption of the barrier produces an increase in TGFβ production and consequent remodelling (Ortiz-Zapater et al., 2022a) or in the gut (Yun et al., 2019), just to name some. Moreover, TGFβ is one of the main communication molecules between the epithelium, immune cells and, especially in the context of ECM and remodelling, fibroblasts. In fact, TGFβ is the main molecule implicated in the differentiation/activation of myofibroblasts (see among many others (Ortiz-Zapater et al., 2022b), the main cell type producing ECM seen in many chronic pathological diseases including asthma and IBD. In that sense, there are numerous studies demonstrating the importance of TGFβ in the asthmatic inflammation and remodelling (Halwani et al., 2011; Al-Alawi et al., 2014) and more recently, it has postulated that the study of TGFβ polymorphisms, in combination with clinical factors, could predict asthma diagnosis with high sensitivity (Panek et al., 2022). In IBD, TGFβ has been studied due to its effect from and towards the epithelium, but also related to the immune response, and curiously, acting directly on the intestinal microbiota (Ihara et al., 2017).

## 4 Asthma

In this review, we have described so far molecules and mechanisms involved in epithelial homeostasis. In the next two sections, we will describe in detail both asthma and IBD as two example diseases where different epithelial driven alterations lead to epithelial barrier dysfunction, highlighting work done in this field and focusing on the epithelium as the potential therapeutic target, alone and in combination with established treatments.

### 4.1 Asthma, the attack, and inflammation

In the second century, Aretaeus of Cappadocia described asthma as *aazein*: a short-drawn breath or panting, a death rattle. Aretaeus went on to describe the defining characteristic of all asthmatics, the attack: “…they open the mouth since no house is sufficient for their respiration, they breathily standing, as if desiring to draw in all the air which they possibly can inhale … ”, and if the symptoms abate, he concludes, “the asthmatic escapes death, but in the intervals between severe attacks or even when they are walking on ground level, they bear in mind the symptoms of the disease (Karamanou and Androutsos, 2011).” The haunting trauma of an asthma attack is echoed in Henry Salter’s account, “…not only is asthma not an uncommon disease, but it is one of the direst suffering; the horrors of the asthmatic paroxysm far exceed any acute bodily pain”. The language defining asthma has remained abstract and scantily more informative through the centuries. And it was not till 2017, when the Global Initiative for Asthma (GINA) defined asthma as, “a heterogenous disease usually characterised by chronic inflammation. It is defined by a history of respiratory symptoms such as wheeze, shortness of breath, chest tightness, and cough that vary over time and in intensity, together with variable expiratory airflow limitations.”

Today, asthma affects more than 300 million people globally, at a staggering financial cost and a burden to quality of life and remains one of the most common, non-communicable diseases (Papi et al., 2018; Pavord et al., 2018; Porsbjerg et al., 2023). Significant advances have been made in asthma care, as hospital admissions and deaths due to asthma are on the decline since the 1990s. The majority of asthma sufferers present with a type 2 inflammatory response and profile characterised by hyper-production of IL4, IL5, and IL13, increased blood eosinophils and fractional exhaled nitric oxide (FeNO) (Porsbjerg et al., 2023). However, current treatments only manage symptoms and have little-to-no effect on the natural progression of this disease. Even the diagnosis and term itself is umbrella, widely understood by clinicians that asthma could represent manifold pulmonary diseases. The use of inhaled corticosteroids became aggressively prescribed in the late 1980s, which resulted in fewer exacerbations and better control of patient symptoms and mortality. This biased physicians and researchers to approach asthma as a chronic inflammatory disorder, where disease symptoms are to be managed but not cured. This “inflammatory-centric” approach was implemented with wilful disregard that asthma attacks, or airway hyperresponsiveness, the sentinel event of all asthmatics, can occur in individuals without inflammation. Further, the degree of inflammation and the types of inflammation effecting asthmatics (eosinophilic, non-eosinophilic, high-type 2, low-type 2, etc.) is well documented to be highly variable (Hammad and Lambrecht, 2021; Porsbjerg et al., 2023). To this, commissions have been gathered to address the problem of asthma, focusing on the outdated thinking and antiquated research practices governing its treatment and prevention (Papi et al., 2018; Pavord et al., 2018; Porsbjerg et al., 2023). Recently, the Lancet compiled a commission to redefine asthma with the aim, “…to identify entrenched areas of asthma management and treatment in which progress has stalled and to challenge current principles … “We believe that the most important cause of this stagnation is a continued reliance on outdated and unhelpful disease labels, treatment and research frameworks, and monitoring strategies, which have reached the stage of unchallenged veneration and have subsequently stifled new thinking (Pavord et al., 2018).”

### 4.2 Epithelial dysregulation and damage in all asthma

It has long been speculated that epithelial loss or damage in asthma studies is due to artefacts from the harvesting and processing protocols while obtaining and analysing tissue samples (e.g., bronchial brushings and biopsies). However, an ever growing number of studies are revealing the loss, damage, and dysregulation of the epithelium in all asthmatics (Payne et al., 2003; Pohunek et al., 2005; van Rijt et al., 2011; Papi et al., 2018; Hammad and Lambrecht, 2021). Loss of the superficial epithelial layer, preferential destruction of ciliated cells, and over expression and activation of EGFR with increases in growth factors, including TGFβ (Hoshino et al., 1998; Shahana et al., 2005; Boxall et al., 2006; Holgate, 2007), are found in the majority of asthma suffers; even occurring in mild, early, and non-fatal asthma. Discussed earlier, damaged epithelium releases a number of soluble mediators promoting remodelling and inflammation (e.g., TSLP, IL25, and IL33), and are not only highly expressed in asthmatic airways, but represent genetic loci identified in a number of genome-wide association (GWA) studies correlating with asthma susceptibility (Cookson, 2004; Allakhverdi et al., 2007; Grotenboer et al., 2013; Moheimani et al., 2016). As an example, Steven Holgate’s group demonstrated that asthmatic children have damaged epithelium with increased expression of EGFR, that was significantly correlated with basement membrane thickness (an important pathological feature of adult asthma), by excessive deposition of collagen III, seen in the absence of eosinophilic inflammation (Fedorov et al., 2005). Moreover, using bronchial biopsies from healthy and asthmatic cohorts, Barbato et al., showed loss of epithelium, increase in angiogenesis, and basement membrane thickening in asthmatic children prior to a mounted inflammatory state (Barbato et al., 2006). In an even earlier study, Marguet and co-workers found increased numbers of epithelial cells in the bronchoalveolar lavage fluid from asthmatic children compared to health controls (Marguet et al., 1999), further suggesting that epithelial loss and damage-not present at birth-is occurring before-or-at disease conception, and likely initiating and sustaining the adaptive response characteristic of most asthmatics.

### 4.3 Barrier dysfunction and asthma

As reviewed before, the epithelium can act as a barrier through the cooperative action of cell junctions with the cytoskeletal apparatus, essential for barrier function and downstream signalling. Dysregulation of the junctions themselves can orchestrate pro-inflammatory signalling pathways, fuelling an inflammatory cascade and feed-forward mechanisms initiated by the wounded barrier. A consequence of barrier damage, is the release of pro-inflammatory factors (e.g. alarmins), known to elicit a type-2 response resulting in increased IL4 and IL13 in airways that are now appreciated to also perpetuate junction dysfunction by downregulation of claudins, occludin, JAM proteins and ZO-1 (Ahdieh et al., 2001; Ortiz-Zapater et al., 2022a). House dust mite (HDM) extract, one of the major causes of asthma (and asthma exacerbations) in children, contains proteases that are known to cleave junctional proteins including occludin and ZO-1, directly participating in barrier dysfunction. Notably, Tan et al., demonstrated that three chronic HDM experimental asthma mouse models, with distinct inflammatory profiles (eosinophilic, neutrophilic, and mixed granulocytic), all had decreased expression of claudin-5, -8, -18, and -23, ZO-1, and occludin, further suggesting that a dysfunctional epithelium is activating and maintaining inflammatory pathologies rather than inflammation as the initial source of epithelial wounding (Tan et al., 2019). This has been recapitulated in human bronchial epithelial cells in air-liquid interface (ALI) culture systems, and bronchial brushings from asthmatic patients. Downregulation of E-cadherin alone resulted in an EGFR-dependent, type 2-biased inflammatory response, and claudin-18 deficiency was demonstrated to promote barrier dysfunction in asthmatic mice and human epithelial cells (Heijink et al., 2007). An ultra-structural analysis of bronchial biopsies of both allergic and non-allergic asthmatics showed junctions and desmosomes damaged, as well as the destruction of ciliated cells in favour of goblet cell hyperplasia and impaired wound healing, with increased basement membrane thickening (Shahana et al., 2005).

The destruction of barrier proteins results in the activation of signalling pathways promoting asthmatic inflammation while directly inhibiting barrier function through the decreased expression of junctional proteins providing a viscous feed-forward cycle of wounding, repair, inflammation, and re-wounding. This highlights the need for therapeutics that are targeted to maintain barrier proteins and function in chronic disease, such as asthma and IBD.

### 4.4 Mechanics effecting epithelium and asthma

There is an established notion that chronic inflammation results in airway hyper-responsiveness, and numerous studies have demonstrated that high doses of oral and inhaled corticosteroids are unable to stop, nor reverse, asthma exacerbations (Childhood Asthma Management Program Research Group et al., 2000; Kips et al., 2000; Guilbert et al., 2006; Porsbjerg et al., 2023). Ultimately, bronchoconstriction is the result of airway remodelling and as we have discussed above, when the epithelium is damaged and junctional proteins disrupted, downstream signalling occurs to respond to assaults; this is true of mechanical forces applied to monolayers. Unique to the lung (and the heart) is that at birth its movements, required for respiration, will not cease until death, causing the lung to be under constant, and constantly changing mechanical forces. Indeed, these forces are required for healthy lung development *in utero* and after birth, and regulated repair responses (Liu et al., 2016; Li et al., 2018). As earlier discussed, mechanical forces govern epithelial numbers within a monolayer. When crowded regions experience compression, unwanted cells are removed by extrusion to regain homeostatic densities, relieving mechanical stresses (Bagley et al., 2023; Eisenhoffer et al., 2012). Airway epithelium during bronchoconstriction will experience dramatic compressive forces, likely causing excessive cell extrusion, damaging the epithelium, while losing barrier function, and promoting further inflammation (Bagley et al., 2023). Importantly, the mechanically-activated protein YAP1 is well-characterized in airway homeostasis and disease, required for proper airway branching (Lin et al., 2017), maintenance, size regulation, and identity of epithelial cells. Mechanical forces are deeply integrated and unavoidably required for all biological aspects needed for lung development, homeostasis, and pathology, and these mechanically-activated epithelial pathways represent a novel, druggable target in wound repair and disease.

The compressive forces applied to the epithelium during an asthma attack is estimated to be about 30 cm H_2_O, at least an order of magnitude greater than the forces felt during normal respiration (Park et al., 2015). Stealing a line from Chris Grainge’s review on airway mechanical compression, “Bronchoconstriction is not only a symptom of asthma but is also a disease modifier” (Veerati et al., 2020). It has now been demonstrated that compressive forces, *in vitro* and *vivo*, lead to expression of genes known to elicit pathological responses in lung disease, including early growth response-1 (EGFR-1), platelet-derived growth factor (PDGF), and TGFβ. Stimulation of repair response pathways through EGFR activation and down-stream signalling, leads to the release of growth factors (e.g., TGFβ) and ECM components (collagens) involved in airway remodelling and disease progression (Ressler et al., 2000). Incubating fibroblasts with conditioned medium from compressed airway epithelial cells resulted in increased collagen deposition, all in the absence of an inflammatory component (Tschumperlin et al., 2003). Park et al. nicely demonstrated that repeated compressive forces alone, over a relatively short time period, could elicit mucus production, e.g., Muc5AC, in normal human bronchiole epithelial cells that was dependent upon EGFR and TGFβ2 (Park and Tschumperlin, 2009). This work was confirmed in humans: volunteers underwent methacholine challenges (only three times over 4 days) to induce bronchoconstriction that lead to increases in TGFβ, collagen, and mucus production in airway epithelial cells, also in the absence of an inflammatory response (Grainge et al., 2011). This is important as mucus hyper-production and secretion remains an intractable problem in many pulmonary disorders, including asthma. Indeed, in a study of 93 fatal asthma cases, near all had mucus obstructions in their airways, where half had more than 80% of airways occluded with mucus plugs (Aegerter and Lambrecht, 2023). Now we appreciate that this mucus problem is not simply a result of goblet cell hyperplasia but also, the expression of mucus in *bona fide* ciliated cells, which is important if we are to develop effective and targeted therapeutics currently missing in today’s clinics. Finally, wounding epithelium itself can induce airway smooth muscle constriction, actively participating in the airway compression-remodelling response. Elegant work by Steven George’s group used *ex vivo* lung slices from rats and laser ablation to destroy signal airway epithelial cells that resulted in a 70% reduction in airway lumens within seconds of cell wounding, followed by further airway smooth muscle contractions over minutes, again in the absence of an inflammatory response by inflammation (Zhou et al., 2012).

### 4.5 The epithelium as a druggable target in asthma

Currently, asthma therapy is big business with annual revenues in the billions, which is on the rise, as all these medications can do is manage symptoms of this common disease, not capable of stopping or reversing its progression. This sentiment is not new, and indeed clinical trials targeting the epithelial-derived alarmins, released by wounded epithelial barriers, have shown promising results. The monoclonal antibody inhibiting TSLP, Tezepelumab, has been demonstrated to significantly supress all three type-2 clinical biomarkers for asthmatics: peripheral blood eosinophils and total IgE (Schleich et al., 2024); while the anti-IL25 and IL33 drugs, Brodalumab and Itepekimab, respectively, were less successful (Chan et al., 2022). Promising work by Wawrzyniak and others in primary human cells were able to reconstitute barrier function, damaged by IL4 and IL13 exposure, from asthmatic patients by inhibiting histone deacetylases (upregulated in asthma), resulting in junctional protein synthesis (Wawrzyniak et al., 2017). Lastly, as we have discussed, mucus is a problem in many pulmonary diseases, and asthma is no exception, with little treatment options available. However, using a mouse model of IL13-induced mucus hyperplasia and primary cells from asthmatics, inhibiting the heat shock protein 90 (HSP90), upregulated in asthma with geldanamycin, blocked, and even reverted, mucus hyperproduction and goblet cell hyperplasia (Pezzulo et al., 2019). In fact, there are ongoing clinical trials for HSP90 inhibitors for various disease (Kitson and Moody, 2013).

To expand upon current asthma treatments and experimental approaches, we need pathophysiologically relevant platforms that allow for efficient and effective drug discovery and development. In the 1990s, Martin Sanders began iconoclastic work in the use of precision cut lung slices (PCLSs) to study lung physiology and pathology (Martin et al., 1996) that has snowballed over the last three decades, as more researchers are being introduced to the power of this *ex vivo* system in basic cell biology and translational studies (Davies et al., 2015; Alsafadi et al., 2017; Huang et al., 2019b; Lam et al., 2023). PCLSs are thin sections of live tissue containing all resident cell-types, while maintaining proper tissue architecture, preserving cell-to-matrix relationships, within complex, interconnected cellular hierarchies, which make up all tissues and organs. *Ex vivo* lung slices have been successfully used in studies from, mice, rats, pigs, sheep, non-human primates, and humans (Alsafadi et al., 2020). They have been used to study airway and arteriole contraction (Martin et al., 1996), tumour biology within intact tissue (Davies et al., 2015), viral infection (Rosales Gerpe et al., 2018), HDM-induced asthma (Ortiz-Zapater et al., 2022a), and fibrosis (Alsafadi et al., 2017), with seemingly endless potential in novel therapeutic development (Liu et al., 2021; Lam et al., 2023). Importantly, the use of *ex vivo* tissue slices reduces the ethical burden for *in vivo* models, because dozens of slices can be obtained from a single lung, decreasing the number of animals needed, and allowing for multiple treatments assessed in a lone animal. And PCLSs are amenable to many live and fixed imaging techniques (including watching an asthma attack in real time), as well as genetic, biochemical, and molecular biology analyses. An important limitation to PCLSs is that viability decreases in culture conditions over time (usually 7–14 days). Therefore, *ex vivo* modelling of chronic diseases or assessing treatments to reverse established pathologies can be limited, requiring the development of better culturing conditions to overcome this problem. Regardless, the power of PCLSs to bridge disease characterization in animal models, and humans, with translational research and positive clinical outcomes is undeniable.

## 5 Inflammatory bowel disease (IBD)

Medical reports from the 17th and 18th century described cases of patients dying after prolonged episodes of diarrhoea, abdominal pain and fever. Later, the first cases of Crohn’s Disease (CD) and Ulcerative Colitis (UC) were described in Great Britain, in 1859 and 1875, respectively. The pathology of UC was firstly described as affecting the mucosa and submucosal of the rectum and extending to the whole colon, featuring a marked infiltration of inflammatory cells, vascular congestion, goblet cell depletion and crypt abscesses (Kirsner and Palmer, 1951). In the case of CD, Warren mentioned, “A progressive sclerosing granulomatous lymphangitis, probably a reaction to an irritative lipid substance in the bowel content.” (Warren and Sommers, 1948), and etiologically associated with microorganisms, abdominal trauma, or impaired vascular/lymphatic circulation. Currently, IBD is used as an over-reaching term to name chronic and relapsing inflammation of the gastrointestinal tract; being CD and UC the most common clinical manifestations. The first epidemiologic approach to study IBD in 1955 initially suggested the impact of the life-style (Melrose, 1955). Ulterior population studies pointed to key epidemiological features of IBD, such as the ethnicity contribution, environment, as well as the familial background. Increasing incidence during the 20^th^ century has been largely seen and presently it is well accepted that IBD has a worldwide distribution, with 6.8 million people being affected in 2017 (GBD, 2017 Inflammatory Bowel Disease Collaborators, 2020).

Most IBD research between the 19th and the 20th centuries was aimed at a differential diagnostic and the development of a therapy in order of improve the life quality of these patients, until the introduction of biological drugs, mainly anti-TNF antibodies. While in the 21st century, researchers focused on the identification of causative pathological mechanisms, which has spotlighted different players leading to the complex breakdown of gut mucosa homeostasis. Currently, IBD is considered a multifactorial disease, which occurs because of an interplay between genetics, environmental and immunological factors, resulting in an uncontrolled immune response against the intestinal microbiome. The complex nature of the disease pathogenesis implies a clear limitation for the development of curative pharmacological treatments, but also highlight the importance of considering alternative approaches. To this, clinicians are beginning to exploit epithelial features for the diagnosis or treatment of IBD patients that have shown promising results, supporting further investigations to understand the causative role of epithelial dysregulation in IBD.

### 5.1 Barrier dysfunction in IBD

In order to maintain tissue homeostasis, the intestinal epithelium acts as a physical and immunological barrier separating the lumen, which contains the microbiota, and the host. “Epithelial leakage”, a common feature in IBD, allows for the invasion of luminal components, which can activate immune cells located at the sub-epithelial space contributing to intestinal inflammation (Martini et al., 2017). Abnormalities in epithelial barrier function can be reflected by an increased permeability, which has been observed in small bowel and colon in CD patients (Jenkins et al., 1988), and has been correlated to the degree of inflammation (Jenkins et al., 1987; Sanderson et al., 1987; Suenaert et al., 2002; Turpin et al., 2020). Moreover, it has been shown that increased intestinal permeability in IBD patients in remission can predict the occurrence of relapse or flares (Wyatt et al., 1993; Irvine and Marshall, 2000; Tibble et al., 2000; Vivinus-Nebot et al., 2014). Together, the occurrence of epithelial barrier dysfunction before the outbreak of the inflammatory response supports the hypothesis of epithelial defects as etiological factors in IBD pathogenesis. GWAS studies identified genes linked to altered barrier function to be associated to IBD; including genetic variants of CARD15/NOD2 gene, resulting in severe forms of CD (D'Inca et al., 2006; Buhner et al., 2006). In fact, several genes relevant to epithelial barrier function have been categorized as IBD loci, such as HFN4, CDH1 and LAMB1 in UC (Consortium et al., 2009). In agreement, several animal models demonstrate that epithelia permeability precedes the development of intestinal inflammation, for example, the IL10 KO (Madsen et al., 1999), and the SAMP/YitFc mouse (Olson et al., 2006), as well as the mouse strain deficient for the xenobiotic transporter mdr1a (Resta-Lenert et al., 2005). The etiological role of epithelial leakage in inflammation is further supported by IBD-like phenotypes in patients suffering from monogenic diseases. For instance, the very-early onset IBD called Tufting enteropathy is caused by mutations in EpCAM, leading to cell-cell contact disruption (Sivagnanam et al., 2008). Although these and other data support the epithelial contribution to the onset and progression of IBD, the low IBD-like phenotype penetrance of these monogenic diseases indicates the existence of functional redundancy between different proteins/pathways within the enterocyte, and/or the requirement for non-epithelial factors for the onset of intestinal inflammation. This idea was indeed confirmed by other mouse models targeting TJ proteins, showing that a leaky barrier is not sufficient to trigger intestinal inflammation, such as JAM-a deficient animals (Khounlotham et al., 2012), or transgenics mice with expression of claudin-2 in IECs (Ahmad et al., 2014).

### 5.2 Epithelial alteration in IBD

As mentioned above, increased epithelial permeability is a hallmark of patients suffering from IBD (Teshima et al., 2012). In 2007, Zeissig *et al.* described upregulation of the pore-formin Claudin-2 and downregulation and/or redistribution of claudin-5, -8 and occludin as the main alterations affecting the apical junctional complex (AJC), and thereby contributing to impaired barrier function in CD (Zeissig et al., 2007). In UC, claudin-2 is also upregulated, while the barrier forming claudin-4 and -7 are downregulated (Oshima et al., 2008), as well as occludin (Heller et al., 2005). In IBD or immune-driven colitis the upregulation of claudin-2 can be attributed, at least partially, to the increased levels of several proinflammatory cyotkines, such as IL13 (Heller et al., 2005). Conversely, Myosin Light Chain Kinase (MLCK) activation causing phosphorylation of MLC and occludin endocytosis contribute to permeability mediated by the leak pathway (Clayburgh et al., 2005; Marchiando et al., 2010; Van Itallie et al., 2010). Previously mentioned, occludin is downregulated in IBD patients (Heller et al., 2005; Kuo et al., 2019), which can be triggered by cytokines such as TNF (Su et al., 2013) or LIGHT (Schwarz et al., 2007). Additionally, the tricellular TJ proteins tricellulin (Krug et al., 2009; Saito et al., 2021) and angulin-1 (Sugawara et al., 2021) also contribute to the leak pathway permeability. Recent studies also pointed to a downregulation of tricellulin expression in UC patients (Krug et al., 2018). Indeed, *in vitro* studies have shown that the pro-inflammatory milieu in the inflamed gut of IBD patients can lead to alterations on several proteins within the AJC, as upon stimulation with IL1β (Al-Sadi et al., 2008), IL6 (Suzuki et al., 2011), IL4 and IL13 (Ceponis et al., 2000), TNF-α (Ma et al., 2004) or IFN-γ (Madara and Stafford, 1989; Wang et al., 2005).

Regulated cytoskeleton function is crucial for TJ assembly and epithelial barrier function, In fact, transcriptional regulation of ACF7, a cytoskeleton crosslinking protein, is observed in UC patients (Ma et al., 2017). Accordingly, mice with an epithelial-specific knockout of non-muscle MyosinIIA suffer from increased intestinal permeability, low scale mucosal inflammation, and increased susceptibility to experimental colitis (Naydenov et al., 2016). Cell stress can also induce changes in actin dynamics and affect actin-binding proteins, such as Villin-1 and Gelsolin, which in turn control survival of Intestinal Epithelial Cells (IECs) and barrier function (Roy et al., 2018). Recent *in vivo* studies demonstrated that prenylation of Rac1 and RhoA, tightly associated to the cytoskeleton, significantly contribute to epithelial barrier function in the gut, and this correlated with alterations of its expression and/or subcellular localization in the intestinal epithelium of IBD patients (Lopez-Posadas et al., 2016; Martinez-Sanchez et al., 2022).

Beyond structural defects, IBD is associated with changes in the epithelial secretome. IBD has been associated to defects of goblet cell differentiation, supporting the key role of the mucus in the intestine (Gersemann et al., 2009). Indeed, the composition of gut mucus is altered in IBD, which is depicted by reduced TFF3, expression diminished levels mucin2 and reduced mucus sulfatation (Tytgat et al., 1996). Focusing on alarmins, TSLP and IL33 have been suggested as protective molecules in IBD (Taylor et al., 2009). Although UC patients show reduced expression of TSLP (Tahaghoghi-Hajghorbani et al., 2019); controversial data are available concerning IL33 in CD and UC (Seidelin et al., 2010; Tahaghoghi-Hajghorbani et al., 2019).

### 5.3 Leaky epithelium as a diagnostic tool

The increasing acceptance of the causative role of epithelial-derived mechanisms in IBD pathogenesis is also reflected by the current effort to exploit this in the clinic, both for treatment and diagnosis of chronic inflammatory diseases. Traditional sugar permeability assays (Meddings and Gibbons, 1998; Teshima and Meddings, 2008) are giving way to molecular imaging, such as confocal-laser endomicroscopy (CLE) using a tracer dye to assess intestinal permeability. This technique permits the identification of epithelial gaps (Kiesslich et al., 2007), even correlating to the occurrence of relapses (Kiesslich et al., 2012) and the identification of subclinical lesions in IBD (Lim et al., 2014; Zaidi et al., 2016). Using this CLE, a recent cross-sectional diagnostic study demonstrated the superiority of barrier healing (*versus* endoscopic/histologic remission) for the prediction of adverse outcomes in CD and UC, validating epithelial leakage as a prognostic marker of the disease (Rath et al., 2023). In order to overcome safety limitations of CLE, a multimodal imaging label-free imaging technique has been used to assess intestinal permeability in UC patients (Quansah et al., 2023). Despite these advanced imaging techniques, there is a clear need for the identification and validation of non-invasive methods for the diagnosis of “leaky gut”. Although several biological markers have been suggested in this context [plasma/serum citrulline, FABP-2, alpha-GST or zonulin; urine claudin-3; or faecal defensins (Bischoff et al., 2014)], none of them has been efficacious in disease prognosis or progression. We believe that the use of CLE (or alternative imaging methods) alone, or in combination with other standard methods (endoscopy/histology), and the identification of biomarkers for impaired intestinal permeability, will allow us to define the functional state of epithelial integrity and contribute to the prediction of IBD flares.

### 5.4 Epithelium as a druggable target in IBD

Currently, the clinical management of IBD strives to control symptoms and mucosal healing (Neurath and Travis, 2012). However, the lack of response to therapy and the low safety profile of immunosuppressive drugs implies the need of alternative therapies, and epithelial restoration emerges as a key component to achieve mucosal healing in IBD, with the final objective of achieving sustained clinical remission, reduced rate of surgery and lower incidence of long term complications. Thus, new knowledge of epithelial dysfunction would likely impact IBD clinical management.

Therapeutic strategies based on promoting the integrity of TJs might have a beneficial effect in IBD. For instance, the zonulin inhibitor AT-1001 (lazarotide) impairs TJ disassembly due to cytoskeleton rearrangement and ameliorates experimental colitis in mice (Arrieta et al., 2009; Sturgeon et al., 2017). Inhibition of the pore function from TJ can limit increased paracellular permeability, which can be achieved for example, by inhibiting casein kinase 2 (Raleigh et al., 2011), indeed providing a certain protection against experimental colitis (Raju et al., 2020). On the other hand, the group of JR. Turner has extensively characterized the MLCK-dependent signaling transduction regulating the leak pathway, culminating in the identification of the small molecule divertin (Graham et al., 2019). Divertin blocks the MLCK1 recruitment via the IgG3 domain to the perijunctional actomyosin ring inhibiting occludin endocytosis and promoting barrier function without altering MLCK enzymatic activity (He et al., 2008; Graham et al., 2019). Strikingly, divertin showed a similar therapeutic effect as anti-TNF in immune-mediated mouse experimental intestinal inflammation (Graham et al., 2019).

The accumulated evidence about the causative role of barrier function in IBD implies the need of assessing the impact of current treatments on the intestinal epithelium, and the potential link to success/lack of response in specific patients. One case in this context is the barrier repair observed upon anti-TNF treatment in CD patients (D'Haens et al., 1999; Rutgeerts et al., 2012; Kierkus et al., 2012), which has been mechanistically linked to the inhibition of IEC apoptosis and Notch pathway modulation (Kawamoto et al., 2019). Additionally, mesalamine treatment improved mucosal healing in clinical trials including mild-to-moderate UC patients (Lichtenstein et al., 2011; Bokemeyer et al., 2012; Probert et al., 2014). In this case, epithelial wound healing can also be promoted by increasing epithelial cell migration and proliferation (Baumgart et al., 2005) and impaired cytokine-driven paracellular permeability (Khare et al., 2019). The beneficial effects of corticosteroids was initially thought to be through the regulation of inflammatory factors (Wild et al., 2003). However, it was recently shown that the exposure of intestinal organoids derived from CD patients to prednisolone rescued the modulated expression/distribution of E-cadherin, ILDR-1, Claudin-2, MLCK and phospho-STAT1 upon cytokine treatment (Xu et al., 2021). Elegant work by Zuo *et al.* described the capacity of tacrolimus to interact with FKBP8, which in turn impairs their interaction with MLCK1 for its recruitment to the acto-myosin ring for the induction of epithelial barrier function (Zuo et al., 2023). Together, this shows the potential contribution of classical immunosuppressive drugs and biologicals to epithelial restoration in the context of IBD.

Many experts have demonstrated that there is a way to confer benefit to the host by administration of probiotics. Probiotics are live organisms that can shape the commensal microbiota and the composition of the mucus. Thus, bacteria such as *Bacillus subtilis* (Li et al., 2020b; Ahl et al., 2016), or *Lactobacillus spp* (Bron et al., 2017) or the *Lactobacillus reuteri* alter mucin production. In fact, *Lactobacillus* showed a protective effect, increasing the mucus layer thickness (Ahl et al., 2016). There are also many different studies reporting the beneficial effects of the supplementation with *A. muciniphila* (Wu et al., 2017; van der Lugt et al., 2019). Moreover, bacteria-derived metabolites altering the mucus composition, such as indoleacrylic acid, have shown protective effects in experimental colitis (Wlodarska et al., 2017). Thus, probiotics and their impact on the mucus layer emerge as interesting mechanisms to impact on intestinal epithelial integrity.

Despite attractive strategies, there is still no pharmacological treatment for epithelial restoration in IBD. This is partially because the limitations for primary intestinal epithelial cultures until the development of intestinal organoids, which impeded the segregation of epithelial intrinsic mechanisms. Organoids are multicellular culture systems embedding in an ECM-like matrix mimicking the 3D architecture of the intestinal epithelium, which are valuable surrogates for intestinal tissue. Importantly, the cellular complexity and plasticity of the intestinal epithelium can also be mimicked in intestinal organoids (Basak et al., 2017; Treveil et al., 2020; Martinez-Silgado et al., 2023), and they can be used for genetic manipulation, biobanking (van de Wetering et al., 2015), and translational studies, since they conserve genetic and epigenetics of the original tissue if derived from ASCs (Dotti et al., 2017). The use of organoids has made possible the validation of molecular signatures linked epithelial alterations in disease (Bigorgne et al., 2014), as well as the identification of new targets in epithelial cell biology with a potential direct application in IBD (Bayrer et al., 2018; Glal et al., 2018; Deuring et al., 2019; Li et al., 2020a). Functionally, permeability assays can be applied to intestinal organoid cultures (Bardenbacher et al., 2020; Rallabandi et al., 2020). For example, the restoration of permeability and remission upon low dose naltrexone organoids studies showed the restoration (Lie et al., 2018), or the cytokine-mediated induction of impaired barrier function in human-derived material (Gleeson et al., 2020). Moreover, the use of organoids has made it possible to study epithelial crosstalk with other players within the intestinal tissue, such as the microbiota (Leber et al., 2018; Roodsant et al., 2020) and immune cells. Thus, co-cultures of mononuclear phagocytes and organoids demonstrated that this intercellular communication is involved in epithelial cell differentiation and can be targetable in IBD (Ihara et al., 2018). Moreover, organoid culture has opened the path to the development of stem cell transplantation therapy to treat refractory ulcers in IBD patients (Yui et al., 2012). Technical development in the field has also allowed to overcome inherent limitations: the use of microinjection into the organoid lumen to study host-microbiota interactions (Saxena et al., 2016); or the inverted polarity of apical-out organoids mimicking the open intestinal lumen (Co et al., 2021). Further, the development of gut-on-a-chip models including non-epithelial cells within the gut tissue, such as the enteric nervous system, the endothelium and immune mediators will for sure have an enormous impact on biomedical research (Shin and Kim, 2022). Altogether, organoids are nowadays an indispensable tool for the development of new therapies in IBD in general, as nicely reviewed by Yoo and Donowitz (Yoo and Donowitz, 2019).

## 6 Concluding remarks

In this review, our aim is to highlight a body of past and present research demonstrating the epithelium’s supremacy in orchestrating all the necessary molecular players and signalling pathways needed to initiate and sustain inflammatory disorders. There is now abundant data to this, clearly demanding a response from researchers, clinicians, and pharmaceutical industries. Using technologies like PCLSs and organoids focusing on the epithelium and its intrinsic pathways and responses will likely produce needed new therapies in chronic inflammatory disorders. Combing these new and evolving epithelial-centric drug targeting strategies with current anti-inflammation treatments could have a powerful impact on the presently situation we find ourselves with chronic disease prevention and progression, especially in asthma and IBD.

## References

[B1] Abdullah L. H. Coakley R. Webster M. J. Zhu Y. Tarran R. Radicioni G. (2018). Mucin production and hydration responses to mucopurulent materials in normal versus cystic fibrosis airway epithelia. Am. J. Respir. Crit. Care Med. 197, 481–491. 10.1164/rccm.201706-1139OC 29099608 PMC5821906

[B2] Aegerter H. Lambrecht B. N. (2023). The pathology of asthma: what is obstructing our view? Annu. Rev. Pathol. 18, 387–409. 10.1146/annurev-pathol-042220-015902 36270294

[B3] Ahdieh M. Vandenbos T. Youakim A. (2001). Lung epithelial barrier function and wound healing are decreased by IL-4 and IL-13 and enhanced by IFN-gamma. Am. J. Physiol. Cell Physiol. 281, C2029–C2038. 10.1152/ajpcell.2001.281.6.C2029 11698262

[B4] Ahl D. Liu H. Schreiber O. Roos S. Phillipson M. Holm L. (2016). Lactobacillus reuteri increases mucus thickness and ameliorates dextran sulphate sodium-induced colitis in mice. Acta Physiol. (Oxf) 217, 300–310. 10.1111/apha.12695 27096537

[B5] Ahmad R. Chaturvedi R. Olivares-Villagomez D. Habib T. Asim M. Shivesh P. (2014). Targeted colonic claudin-2 expression renders resistance to epithelial injury, induces immune suppression, and protects from colitis. Mucosal Immunol. 7, 1340–1353. 10.1038/mi.2014.21 24670427 PMC4221190

[B6] Al-Alawi M. Hassan T. Chotirmall S. H. (2014). Transforming growth factor β and severe asthma: a perfect storm. Respir. Med. 108, 1409–1423. 10.1016/j.rmed.2014.08.008 25240764

[B7] Ali S. Mohs A. Thomas M. Klare J. Ross R. Schmitz M. L. (2011). The dual function cytokine IL-33 interacts with the transcription factor NF-κB to dampen NF-κB-stimulated gene transcription. J. Immunol. 187, 1609–1616. 10.4049/jimmunol.1003080 21734074

[B8] Allakhverdi Z. Comeau M. R. Jessup H. K. Yoon B. R. Brewer A. Chartier S. (2007). Thymic stromal lymphopoietin is released by human epithelial cells in response to microbes, trauma, or inflammation and potently activates mast cells. J. Exp. Med. 204, 253–258. 10.1084/jem.20062211 17242164 PMC2118732

[B9] Al-Sadi R. Ye D. Dokladny K. Ma T. Y. (2008). Mechanism of IL-1beta-induced increase in intestinal epithelial tight junction permeability. J. Immunol. 180, 5653–5661. 10.4049/jimmunol.180.8.5653 18390750 PMC3035485

[B10] Alsafadi H. N. Staab-Weijnitz C. A. Lehmann M. Lindner M. Peschel B. Konigshoff M. (2017). An *ex vivo* model to induce early fibrosis-like changes in human precision-cut lung slices. Am. J. Physiol. Lung Cell Mol. Physiol. 312, L896–L902. 10.1152/ajplung.00084.2017 28314802

[B11] Alsafadi H. N. Uhl F. E. Pineda R. H. Bailey K. E. Rojas M. Wagner D. E. (2020). Applications and approaches for three-dimensional precision-cut lung slices. Disease modeling and drug discovery. Am. J. Respir. Cell Mol. Biol. 62, 681–691. 10.1165/rcmb.2019-0276TR 31991090 PMC7401444

[B12] Alvarez Y. Smutny M. (2022). Emerging role of mechanical forces in cell fate acquisition. Front. Cell Dev. Biol. 10, 864522. 10.3389/fcell.2022.864522 35676934 PMC9168747

[B13] Angulo-Urarte A. Van Der Wal T. Huveneers S. (2020). Cell-cell junctions as sensors and transducers of mechanical forces. Biochim. Biophys. Acta Biomembr. 1862, 183316. 10.1016/j.bbamem.2020.183316 32360073

[B14] Archer N. K. Jo J. H. Lee S. K. Kim D. Smith B. Ortines R. V. (2019). Injury, dysbiosis, and filaggrin deficiency drive skin inflammation through keratinocyte IL-1α release. J. Allergy Clin. Immunol. 143, 1426–1443 e6. 10.1016/j.jaci.2018.08.042 30240702 PMC6424655

[B15] Arikoglu T. Akyilmaz E. Yildirim D. D. Batmaz S. B. Ulger S. T. Aslan G. (2017). The relation of innate and adaptive immunity with viral-induced acute asthma attacks: focusing on IP-10 and cathelicidin. Allergol. Immunopathol. Madr. 45, 160–168. 10.1016/j.aller.2016.07.003 27955890 PMC7126540

[B16] Arrieta M. C. Madsen K. Doyle J. Meddings J. (2009). Reducing small intestinal permeability attenuates colitis in the IL10 gene-deficient mouse. Gut 58, 41–48. 10.1136/gut.2008.150888 18829978 PMC2597688

[B17] Bagley D. C. Russell T. Ortiz-Zapater E. Fox K. Redd P. F. Joseph M. (2023). Bronchoconstriction damages airway epithelia by excess crowding-induced extrusion. bioRxiv, 2023.08.04.551943. 2023.08.04.551943. 10.1101/2023.08.04.551943 PMC7619646 38574138

[B18] Barbato A. Turato G. Baraldo S. Bazzan E. Calabrese F. Panizzolo C. (2006). Epithelial damage and angiogenesis in the airways of children with asthma. Am. J. Respir. Crit. Care Med. 174, 975–981. 10.1164/rccm.200602-189OC 16917118

[B19] Bardenbacher M. Ruder B. Britzen-Laurent N. Naschberger E. Becker C. Palmisano R. (2020). Investigating intestinal barrier breakdown in living organoids. J. Vis. Exp. 10.3791/60546 32281970

[B20] Barry E. R. Morikawa T. Butler B. L. Shrestha K. De La Rosa R. Yan K. S. (2013). Restriction of intestinal stem cell expansion and the regenerative response by YAP. Nature 493, 106–110. 10.1038/nature11693 23178811 PMC3536889

[B21] Basak O. Beumer J. Wiebrands K. Seno H. Van Oudenaarden A. Clevers H. (2017). Induced quiescence of Lgr5+ stem cells in intestinal organoids enables differentiation of hormone-producing enteroendocrine cells. Cell Stem Cell 20, 177–190. 10.1016/j.stem.2016.11.001 27939219

[B22] Bastounis E. E. Serrano-Alcalde F. Radhakrishnan P. Engstrom P. Gomez-Benito M. J. Oswald M. S. (2021). Mechanical competition triggered by innate immune signaling drives the collective extrusion of bacterially infected epithelial cells. Dev. Cell 56, 443–460 e11. 10.1016/j.devcel.2021.01.012 33621492 PMC7982222

[B23] Bauche D. Marie J. C. (2017). Transforming growth factor β: a master regulator of the gut microbiota and immune cell interactions. Clin. Transl. Immunol. 6, e136. 10.1038/cti.2017.9 PMC5418590 28523126

[B24] Baumgart D. C. Vierziger K. Sturm A. Wiedenmann B. Dignass A. U. (2005). Mesalamine promotes intestinal epithelial wound healing *in vitro* through a TGF-beta-independent mechanism. Scand. J. Gastroenterol. 40, 958–964. 10.1080/00365520510015854 16165710

[B25] Bayrer J. R. Wang H. Nattiv R. Suzawa M. Escusa H. S. Fletterick R. J. (2018). LRH-1 mitigates intestinal inflammatory disease by maintaining epithelial homeostasis and cell survival. Nat. Commun. 9, 4055. 10.1038/s41467-018-06137-w 30305617 PMC6180039

[B26] Becker C. Neurath M. F. Wirtz S. (2015). The intestinal microbiota in inflammatory bowel disease. ILAR J. 56, 192–204. 10.1093/ilar/ilv030 26323629

[B27] Benham-Pyle B. W. Pruitt B. L. Nelson W. J. (2015). Cell adhesion. Mechanical strain induces E-cadherin-dependent Yap1 and β-catenin activation to drive cell cycle entry. Science 348, 1024–1027. 10.1126/science.aaa4559 26023140 PMC4572847

[B28] Benjamin J. Makharia G. K. Ahuja V. Kalaivani M. Joshi Y. K. (2008). Intestinal permeability and its association with the patient and disease characteristics in Crohn's disease. World J. Gastroenterol. 14, 1399–1405. 10.3748/wjg.14.1399 18322955 PMC2693689

[B29] Bergstrom K. S. Xia L. (2013). Mucin-type O-glycans and their roles in intestinal homeostasis. Glycobiology 23, 1026–1037. 10.1093/glycob/cwt045 23752712 PMC3858029

[B30] Beumer J. Artegiani B. Post Y. Reimann F. Gribble F. Nguyen T. N. (2018). Enteroendocrine cells switch hormone expression along the crypt-to-villus BMP signalling gradient. Nat. Cell Biol. 20, 909–916. 10.1038/s41556-018-0143-y 30038251 PMC6276989

[B31] Beumer J. Clevers H. (2021). Cell fate specification and differentiation in the adult mammalian intestine. Nat. Rev. Mol. Cell Biol. 22, 39–53. 10.1038/s41580-020-0278-0 32958874

[B32] Beutel O. Maraspini R. Pombo-Garcia K. Martin-Lemaitre C. Honigmann A. (2019). Phase separation of zonula occludens proteins drives formation of tight junctions. Cell 179, 923–936. 10.1016/j.cell.2019.10.011 31675499

[B33] Bigorgne A. E. Farin H. F. Lemoine R. Mahlaoui N. Lambert N. Gil M. (2014). TTC7A mutations disrupt intestinal epithelial apicobasal polarity. J. Clin. Invest. 124, 328–337. 10.1172/JCI71471 24292712 PMC3871247

[B34] Bischoff S. C. Barbara G. Buurman W. Ockhuizen T. Schulzke J. D. Serino M. (2014). Intestinal permeability--a new target for disease prevention and therapy. BMC Gastroenterol. 14, 189. 10.1186/s12876-014-0189-7 25407511 PMC4253991

[B35] Bokemeyer B. Hommes D. Gill I. Broberg P. Dignass A. (2012). Mesalazine in left-sided ulcerative colitis: efficacy analyses from the PODIUM trial on maintenance of remission and mucosal healing. J. Crohns Colitis 6, 476–482. 10.1016/j.crohns.2011.10.006 22398060

[B36] Borchers N. S. Santos-Valente E. Toncheva A. A. Wehkamp J. Franke A. Gaertner V. D. (2021). Human β-defensin 2 mutations are associated with asthma and atopy in children and its application prevents atopic asthma in a mouse model. Front. Immunol. 12, 636061. 10.3389/fimmu.2021.636061 33717182 PMC7946850

[B37] Boxall C. Holgate S. T. Davies D. E. (2006). The contribution of transforming growth factor-beta and epidermal growth factor signalling to airway remodelling in chronic asthma. Eur. Respir. J. 27, 208–229. 10.1183/09031936.06.00130004 16387953

[B38] Braga V. (2018). Signaling by small GTPases at cell-cell junctions: protein interactions building control and networks. Cold Spring Harb. Perspect. Biol. 10, a028746. 10.1101/cshperspect.a028746 28893858 PMC6169809

[B39] Bron P. A. Kleerebezem M. Brummer R. J. Cani P. D. Mercenier A. Macdonald T. T. (2017). Can probiotics modulate human disease by impacting intestinal barrier function? Br. J. Nutr. 117, 93–107. 10.1017/S0007114516004037 28102115 PMC5297585

[B40] Buhner S. Buning C. Genschel J. Kling K. Herrmann D. Dignass A. (2006). Genetic basis for increased intestinal permeability in families with Crohn's disease: role of CARD15 3020insC mutation? Gut 55, 342–347. 10.1136/gut.2005.065557 16000642 PMC1856071

[B41] Burclaff J. Bliton R. J. Breau K. A. Ok M. T. Gomez-Martinez I. Ranek J. S. (2022). A proximal-to-distal survey of healthy adult human small intestine and colon epithelium by single-cell transcriptomics. Cell Mol. Gastroenterol. Hepatol. 13, 1554–1589. 10.1016/j.jcmgh.2022.02.007 35176508 PMC9043569

[B42] Calven J. Ax E. Radinger M. (2020). The airway epithelium-A central player in asthma pathogenesis. Int. J. Mol. Sci. 21, 8907. 10.3390/ijms21238907 33255348 PMC7727704

[B43] Camargo F. D. Gokhale S. Johnnidis J. B. Fu D. Bell G. W. Jaenisch R. (2007). YAP1 increases organ size and expands undifferentiated progenitor cells. Curr. Biol. 17, 2054–2060. 10.1016/j.cub.2007.10.039 17980593

[B44] Ceponis P. J. Botelho F. Richards C. D. Mckay D. M. (2000). Interleukins 4 and 13 increase intestinal epithelial permeability by a phosphatidylinositol 3-kinase pathway. Lack of evidence for STAT 6 involvement. J. Biol. Chem. 275, 29132–29137. 10.1074/jbc.M003516200 10871612

[B45] Chan R. Stewart K. Misirovs R. Lipworth B. J. (2022). Targeting downstream type 2 cytokines or upstream epithelial alarmins for severe asthma. J. Allergy Clin. Immunol. Pract. 10, 1497–1505. 10.1016/j.jaip.2022.01.040 35131510

[B46] Chen L. Brenner D. A. Kisseleva T. (2019). Combatting fibrosis: exosome‐based therapies in the regression of liver fibrosis. Hepatol. Commun. 3, 180–192. 10.1002/hep4.1290 30766956 PMC6357832

[B47] Childhood Asthma Management Program Research Group Szefler S. Weiss S. Tonascia J. Adkinson N. F. Bender B. (2000). Long-term effects of budesonide or nedocromil in children with asthma. N. Engl. J. Med. 343, 1054–1063. 10.1056/NEJM200010123431501 11027739

[B48] Citi S. Guerrera D. Spadaro D. Shah J. (2014). Epithelial junctions and Rho family GTPases: the zonular signalosome. Small GTPases 5, 1–15. 10.4161/21541248.2014.973760 PMC4601189 25483301

[B49] Clancy D. M. Sullivan G. P. Moran H. B. T. Henry C. M. Reeves E. P. Mcelvaney N. G. (2018). Extracellular neutrophil proteases are efficient regulators of IL-1, IL-33, and IL-36 cytokine activity but poor effectors of microbial killing. Cell Rep. 22, 2937–2950. 10.1016/j.celrep.2018.02.062 29539422

[B50] Clayburgh D. R. Barrett T. A. Tang Y. Meddings J. B. Van Eldik L. J. Watterson D. M. (2005). Epithelial myosin light chain kinase-dependent barrier dysfunction mediates T cell activation-induced diarrhea *in vivo* . J. Clin. Invest. 115, 2702–2715. 10.1172/JCI24970 16184195 PMC1224297

[B51] Co J. Y. Margalef-Catala M. Monack D. M. Amieva M. R. (2021). Controlling the polarity of human gastrointestinal organoids to investigate epithelial biology and infectious diseases. Nat. Protoc. 16, 5171–5192. 10.1038/s41596-021-00607-0 34663962 PMC8841224

[B52] Consortium U. I. G. Barrett J. C. Lee J. C. Lees C. W. Prescott N. J. Anderson C. A. (2009). Genome-wide association study of ulcerative colitis identifies three new susceptibility loci, including the HNF4A region. Nat. Genet. 41, 1330–1334. 10.1038/ng.483 19915572 PMC2812019

[B53] Cookson W. (2004). The immunogenetics of asthma and eczema: a new focus on the epithelium. Nat. Rev. Immunol. 4, 978–988. 10.1038/nri1500 15573132

[B54] Cornick S. Tawiah A. Chadee K. (2015). Roles and regulation of the mucus barrier in the gut. Tissue Barriers 3, e982426. 10.4161/21688370.2014.982426 25838985 PMC4372027

[B55] Davies E. J. Dong M. Gutekunst M. Narhi K. Van Zoggel H. J. Blom S. (2015). Capturing complex tumour biology *in vitro*: histological and molecular characterisation of precision cut slices. Sci. Rep. 5, 17187. 10.1038/srep17187 26647838 PMC4673528

[B56] Deprez M. Zaragosi L. E. Truchi M. Becavin C. Ruiz Garcia S. Arguel M. J. (2020). A single-cell atlas of the human healthy airways. Am. J. Respir. Crit. Care Med. 202, 1636–1645. 10.1164/rccm.201911-2199OC 32726565

[B57] Deuring J. J. Li M. Cao W. Chen S. Wang W. De Haar C. (2019). Pregnane X receptor activation constrains mucosal NF-κB activity in active inflammatory bowel disease. PLoS One 14, e0221924. 10.1371/journal.pone.0221924 31581194 PMC6776398

[B58] D'Haens G. Van Deventer S. Van Hogezand R. Chalmers D. Kothe C. Baert F. (1999). Endoscopic and histological healing with infliximab anti-tumor necrosis factor antibodies in Crohn's disease: a European multicenter trial. Gastroenterology 116, 1029–1034. 10.1016/s0016-5085(99)70005-3 10220494

[B59] D'Inca R. Annese V. Di Leo V. Latiano A. Quaino V. Abazia C. (2006). Increased intestinal permeability and NOD2 variants in familial and sporadic Crohn's disease. Aliment. Pharmacol. Ther. 23, 1455–1461. 10.1111/j.1365-2036.2006.02916.x 16669960

[B60] Dotti I. Mora-Buch R. Ferrer-Picon E. Planell N. Jung P. Masamunt M. C. (2017). Alterations in the epithelial stem cell compartment could contribute to permanent changes in the mucosa of patients with ulcerative colitis. Gut 66, 2069–2079. 10.1136/gutjnl-2016-312609 27803115 PMC5749340

[B61] Duckworth C. A. (2021). Identifying key regulators of the intestinal stem cell niche. Biochem. Soc. Trans. 49, 2163–2176. 10.1042/BST20210223 34665221 PMC8589435

[B62] Eisenhoffer G. T. Loftus P. D. Yoshigi M. Otsuna H. Chien C. B. Morcos P. A. (2012). Crowding induces live cell extrusion to maintain homeostatic cell numbers in epithelia. Nature 484, 546–549. 10.1038/nature10999 22504183 PMC4593481

[B63] Elmentaite R. Kumasaka N. Roberts K. Fleming A. Dann E. King H. W. (2021). Cells of the human intestinal tract mapped across space and time. Nature 597, 250–255. 10.1038/s41586-021-03852-1 34497389 PMC8426186

[B64] Elphick D. Liddell S. Mahida Y. R. (2008). Impaired luminal processing of human defensin-5 in Crohn’s disease: persistence in a complex with chymotrypsinogen and trypsin. Am. J. Pathol. 172, 702–713. 10.2353/ajpath.2008.070755 18258845 PMC2258249

[B65] Etienne-Mesmin L. Chassaing B. Desvaux M. De Paepe K. Gresse R. Sauvaitre T. (2019). Experimental models to study intestinal microbes–mucus interactions in health and disease. FEMS Microbiol. Rev. 43, 457–489. 10.1093/femsre/fuz013 31162610

[B66] Fahy J. V. Steiger D. J. Liu J. Basbaum C. B. Finkbeiner W. E. Boushey H. A. (1993). Markers of mucus secretion and DNA levels in induced sputum from asthmatic and from healthy subjects. Am. Rev. Respir. Dis. 147, 1132–1137. 10.1164/ajrccm/147.5.1132 8484621

[B67] Fedorov I. A. Wilson S. J. Davies D. E. Holgate S. T. (2005). Epithelial stress and structural remodelling in childhood asthma. Thorax 60, 389–394. 10.1136/thx.2004.030262 15860714 PMC1758889

[B68] Fellermann K. Stange D. E. Schaeffeler E. Schmalzl H. Wehkamp J. Bevins C. L. (2006). A chromosome 8 gene-cluster polymorphism with low human beta-defensin 2 gene copy number predisposes to Crohn disease of the colon. Am. J. Hum. Genet. 79, 439–448. 10.1086/505915 16909382 PMC1559531

[B69] Forbes J. D. Van Domselaar G. Bernstein C. N. (2016). Microbiome survey of the inflamed and noninflamed gut at different compartments within the gastrointestinal tract of inflammatory bowel disease patients. Inflamm. Bowel Dis. 22, 817–825. 10.1097/MIB.0000000000000684 26937623

[B70] Fornasa G. Tsilingiri K. Caprioli F. Botti F. Mapelli M. Meller S. (2015). Dichotomy of short and long thymic stromal lymphopoietin isoforms in inflammatory disorders of the bowel and skin. J. Allergy Clin. Immunol. 136, 413–422. 10.1016/j.jaci.2015.04.011 26014813 PMC4534776

[B71] Fre S. Huyghe M. Mourikis P. Robine S. Louvard D. Artavanis-Tsakonas S. (2005). Notch signals control the fate of immature progenitor cells in the intestine. Nature 435, 964–968. 10.1038/nature03589 15959516

[B72] Ganesan S. Comstock A. T. Sajjan U. S. (2013). Barrier function of airway tract epithelium. Tissue Barriers 1, e24997. 10.4161/tisb.24997 24665407 PMC3783221

[B73] Gao W. Li L. Wang Y. Zhang S. Adcock I. M. Barnes P. J. (2015). Bronchial epithelial cells: the key effector cells in the pathogenesis of chronic obstructive pulmonary disease? Respirology 20, 722–729. 10.1111/resp.12542 25868842

[B74] Garabedian E. M. Roberts L. J. Mcnevin M. S. Gordon J. I. (1997). Examining the role of Paneth cells in the small intestine by lineage ablation in transgenic mice. J. Biol. Chem. 272, 23729–23740. 10.1074/jbc.272.38.23729 9295317

[B75] Garcia M. A. Nelson W. J. Chavez N. (2018). Cell-cell junctions organize structural and signaling networks. Cold Spring Harb. Perspect. Biol. 10, a029181. 10.1101/cshperspect.a029181 28600395 PMC5773398

[B76] GBD 2017 Inflammatory Bowel Disease Collaborators (2020). The global, regional, and national burden of inflammatory bowel disease in 195 countries and territories, 1990-2017: a systematic analysis for the Global Burden of Disease Study 2017. Lancet Gastroenterol. Hepatol. 5, 17–30. 10.1016/S2468-1253(19)30333-4 31648971 PMC7026709

[B77] Gersemann M. Becker S. Kubler I. Koslowski M. Wang G. Herrlinger K. R. (2009). Differences in goblet cell differentiation between Crohn's disease and ulcerative colitis. Differentiation 77, 84–94. 10.1016/j.diff.2008.09.008 19281767

[B78] Glal D. Sudhakar J. N. Lu H. H. Liu M. C. Chiang H. Y. Liu Y. C. (2018). ATF3 sustains IL-22-induced STAT3 phosphorylation to maintain mucosal immunity through inhibiting phosphatases. Front. Immunol. 9, 2522. 10.3389/fimmu.2018.02522 30455690 PMC6230592

[B79] Gleeson J. P. Estrada H. Q. Yamashita M. Svendsen C. N. Targan S. R. Barrett R. J. (2020). Development of physiologically responsive human iPSC-derived intestinal epithelium to study barrier dysfunction in IBD. Int. J. Mol. Sci. 21, 1438. 10.3390/ijms21041438 32093254 PMC7073090

[B80] Goodwin K. Nelson C. M. (2021). Mechanics of development. Dev. Cell 56, 240–250. 10.1016/j.devcel.2020.11.025 33321105 PMC8177046

[B81] Goswami S. Angkasekwinai P. Shan M. Greenlee K. J. Barranco W. T. Polikepahad S. (2009). Divergent functions for airway epithelial matrix metalloproteinase 7 and retinoic acid in experimental asthma. Nat. Immunol. 10, 496–503. 10.1038/ni.1719 19329997 PMC5298936

[B82] Graham W. V. He W. Marchiando A. M. Zha J. Singh G. Li H. S. (2019). Intracellular MLCK1 diversion reverses barrier loss to restore mucosal homeostasis. Nat. Med. 25, 690–700. 10.1038/s41591-019-0393-7 30936544 PMC6461392

[B83] Grainge C. L. Lau L. C. Ward J. A. Dulay V. Lahiff G. Wilson S. (2011). Effect of bronchoconstriction on airway remodeling in asthma. N. Engl. J. Med. 364, 2006–2015. 10.1056/NEJMoa1014350 21612469

[B84] Grotenboer N. S. Ketelaar M. E. Koppelman G. H. Nawijn M. C. (2013). Decoding asthma: translating genetic variation in IL33 and IL1RL1 into disease pathophysiology. J. Allergy Clin. Immunol. 131, 856–865. 10.1016/j.jaci.2012.11.028 23380221

[B85] Gudipaty S. A. Lindblom J. Loftus P. D. Redd M. J. Edes K. Davey C. F. (2017). Mechanical stretch triggers rapid epithelial cell division through Piezo1. Nature 543, 118–121. 10.1038/nature21407 28199303 PMC5334365

[B86] Guilbert T. W. Morgan W. J. Zeiger R. S. Mauger D. T. Boehmer S. J. Szefler S. J. (2006). Long-term inhaled corticosteroids in preschool children at high risk for asthma. N. Engl. J. Med. 354, 1985–1997. 10.1056/NEJMoa051378 16687711

[B87] Gustafsson J. K. Ermund A. Ambort D. Johansson M. E. Nilsson H. E. Thorell K. (2012). Bicarbonate and functional CFTR channel are required for proper mucin secretion and link cystic fibrosis with its mucus phenotype. J. Exp. Med. 209, 1263–1272. 10.1084/jem.20120562 22711878 PMC3405509

[B88] Haas A. J. Zihni C. Krug S. M. Maraspini R. Otani T. Furuse M. (2022). ZO-1 guides tight junction assembly and epithelial morphogenesis via cytoskeletal tension-dependent and -independent functions. Cells 11, 3775. 10.3390/cells11233775 36497035 PMC9740252

[B89] Haber A. L. Biton M. Rogel N. Herbst R. H. Shekhar K. Smillie C. (2017). A single-cell survey of the small intestinal epithelium. Nature 551, 333–339. 10.1038/nature24489 29144463 PMC6022292

[B90] Halim T. Y. Steer C. A. Matha L. Gold M. J. Martinez-Gonzalez I. Mcnagny K. M. (2014). Group 2 innate lymphoid cells are critical for the initiation of adaptive T helper 2 cell-mediated allergic lung inflammation. Immunity 40, 425–435. 10.1016/j.immuni.2014.01.011 24613091 PMC4210641

[B91] Halwani R. Al-Muhsen S. Al-Jahdali H. Hamid Q. (2011). Role of transforming growth factor-β in airway remodeling in asthma. Am. J. Respir. Cell Mol. Biol. 44, 127–133. 10.1165/rcmb.2010-0027TR 20525803

[B92] Ham J. Shin J. W. Ko B. C. Kim H. Y. (2022). Targeting the epithelium-derived innate cytokines: from bench to bedside. Immune Netw. 22, e11. 10.4110/in.2022.22.e11 35291657 PMC8901708

[B93] Hammad H. Lambrecht B. N. (2015). Barrier epithelial cells and the control of type 2 immunity. Immunity 43, 29–40. 10.1016/j.immuni.2015.07.007 26200011

[B94] Hammad H. Lambrecht B. N. (2021). The basic immunology of asthma. Cell 184, 1469–1485. 10.1016/j.cell.2021.02.016 33711259

[B95] Haniffa M. Taylor D. Linnarsson S. Aronow B. J. Bader G. D. Barker R. A. (2021). A roadmap for the human developmental cell atlas. Nature 597, 196–205. 10.1038/s41586-021-03620-1 34497388 PMC10337595

[B96] Hansson G. C. (2019). Mucus and mucins in diseases of the intestinal and respiratory tracts. J. Intern Med. 285, 479–490. 10.1111/joim.12910 30963635 PMC6497544

[B97] He W. Q. Peng Y. J. Zhang W. C. Lv N. Tang J. Chen C. (2008). Myosin light chain kinase is central to smooth muscle contraction and required for gastrointestinal motility in mice. Gastroenterology 135, 610–620. 10.1053/j.gastro.2008.05.032 18586037 PMC2648853

[B98] Heijink I. H. Kies P. M. Kauffman H. F. Postma D. S. Van Oosterhout A. J. Vellenga E. (2007). Down-regulation of E-cadherin in human bronchial epithelial cells leads to epidermal growth factor receptor-dependent Th2 cell-promoting activity. J. Immunol. 178, 7678–7685. 10.4049/jimmunol.178.12.7678 17548604

[B99] Heller F. Florian P. Bojarski C. Richter J. Christ M. Hillenbrand B. (2005). Interleukin-13 is the key effector Th2 cytokine in ulcerative colitis that affects epithelial tight junctions, apoptosis, and cell restitution. Gastroenterology 129, 550–564. 10.1016/j.gastro.2005.05.002 16083712

[B100] Henderson A. G. Ehre C. Button B. Abdullah L. H. Cai L. H. Leigh M. W. (2014). Cystic fibrosis airway secretions exhibit mucin hyperconcentration and increased osmotic pressure. J. Clin. Invest. 124, 3047–3060. 10.1172/JCI73469 24892808 PMC4072023

[B101] He S. Lei P. Kang W. Cheung P. Xu T. Mana M. (2023). Stiffness restricts the stemness of the intestinal stem cells and skews their differentiation toward goblet cells. Gastroenterology 164, 1137–1151 e15. 10.1053/j.gastro.2023.02.030 36871599 PMC10200762

[B102] Hicks-Berthet J. Ning B. Federico A. Tilston-Lunel A. Matschulat A. Ai X. (2021). Yap/Taz inhibit goblet cell fate to maintain lung epithelial homeostasis. Cell Rep. 36, 109347. 10.1016/j.celrep.2021.109347 34260916 PMC8346236

[B103] Hiemstra P. (2006). DEFENSINS. Encyclopedia of respiratory medicine. Elsevier. Internet 2006 cited 2023 May 31.

[B104] Hill D. B. Long R. F. Kissner W. J. Atieh E. Garbarine I. C. Markovetz M. R. (2018). Pathological mucus and impaired mucus clearance in cystic fibrosis patients result from increased concentration, not altered pH. Eur. Respir. J. 52, 1801297. 10.1183/13993003.01297-2018 30361244 PMC6446239

[B105] Hippee C. E. Singh B. K. Thurman A. L. Cooney A. L. Pezzulo A. A. Cattaneo R. (2021). Measles virus exits human airway epithelia within dislodged metabolically active infectious centers. PLoS Pathog. 17, e1009458. 10.1371/journal.ppat.1009458 34383863 PMC8384213

[B106] Holgate S. T. (2007). Epithelium dysfunction in asthma. J. Allergy Clin. Immunol. 120, 1233–1244. quiz 1245-6. 10.1016/j.jaci.2007.10.025 18073119

[B107] Horowitz A. Chanez-Paredes S. D. Haest X. Turner J. R. (2023). Paracellular permeability and tight junction regulation in gut health and disease. Nat. Rev. Gastroenterol. Hepatol. 20, 417–432. 10.1038/s41575-023-00766-3 37186118 PMC10127193

[B108] Ho S. Pothoulakis C. Koon H. W. (2013). Antimicrobial peptides and colitis. Curr. Pharm. Des. 19, 40–47. 10.2174/13816128130108 22950497 PMC3662473

[B109] Hoshino M. Nakamura Y. Sim J. J. (1998). Expression of growth factors and remodelling of the airway wall in bronchial asthma. Thorax 53, 21–27. 10.1136/thx.53.1.21 9577517 PMC1758699

[B110] Huang B. Chen Z. Geng L. Wang J. Liang H. Cao Y. (2019a). Mucosal profiling of pediatric-onset colitis and IBD reveals common pathogenics and therapeutic pathways. Cell 179, 1160–1176. 10.1016/j.cell.2019.10.027 31730855

[B111] Huang S. Chen B. Humeres C. Alex L. Hanna A. Frangogiannis N. G. (2020). The role of Smad2 and Smad3 in regulating homeostatic functions of fibroblasts *in vitro* and in adult mice. Biochim. Biophys. Acta Mol. Cell Res. 1867, 118703. 10.1016/j.bbamcr.2020.118703 32179057 PMC7261645

[B112] Huang X. Li L. Ammar R. Zhang Y. Wang Y. Ravi K. (2019b). Molecular characterization of a precision-cut rat lung slice model for the evaluation of antifibrotic drugs. Am. J. Physiol. Lung Cell Mol. Physiol. 316, L348–L357. 10.1152/ajplung.00339.2018 30489156

[B113] Huang Y. Guo L. Qiu J. Chen X. Hu-Li J. Siebenlist U. (2015). IL-25-responsive, lineage-negative KLRG1(hi) cells are multipotential 'inflammatory' type 2 innate lymphoid cells. Nat. Immunol. 16, 161–169. 10.1038/ni.3078 25531830 PMC4297567

[B114] Ihara S. Hirata Y. Hikiba Y. Yamashita A. Tsuboi M. Hata M. (2018). Adhesive interactions between mononuclear phagocytes and intestinal epithelium perturb normal epithelial differentiation and serve as a therapeutic target in inflammatory bowel disease. J. Crohns Colitis 12, 1219–1231. 10.1093/ecco-jcc/jjy088 29917067

[B115] Ihara S. Hirata Y. Koike K. (2017). TGF-β in inflammatory bowel disease: a key regulator of immune cells, epithelium, and the intestinal microbiota. J. Gastroenterol. 52, 777–787. 10.1007/s00535-017-1350-1 28534191

[B116] Ikeda K. Nakajima H. Suzuki K. Kagami S. Hirose K. Suto A. (2003). Mast cells produce interleukin-25 upon Fc epsilon RI-mediated activation. Blood 101, 3594–3596. 10.1182/blood-2002-09-2817 12511410

[B117] Irvine E. J. Marshall J. K. (2000). Increased intestinal permeability precedes the onset of Crohn's disease in a subject with familial risk. Gastroenterology 119, 1740–1744. 10.1053/gast.2000.20231 11113095

[B118] Jackson N. D. Everman J. L. Chioccioli M. Feriani L. Goldfarbmuren K. C. Sajuthi S. P. (2020). Single-cell and population transcriptomics reveal pan-epithelial remodeling in type 2-high asthma. Cell Rep. 32, 107872. 10.1016/j.celrep.2020.107872 32640237 PMC8046336

[B119] Jenkins R. T. Jones D. B. Goodacre R. L. Collins S. M. Coates G. Hunt R. H. (1987). Reversibility of increased intestinal permeability to 51Cr-EDTA in patients with gastrointestinal inflammatory diseases. Am. J. Gastroenterol. 82, 1159–1164. 3118697

[B120] Jenkins R. T. Ramage J. K. Jones D. B. Collins S. M. Goodacre R. L. Hunt R. H. (1988). Small bowel and colonic permeability to 51Cr-EDTA in patients with active inflammatory bowel disease. Clin. Invest. Med. 11, 151–155. 3135136

[B121] Johansson M. E. Sjovall H. Hansson G. C. (2013). The gastrointestinal mucus system in health and disease. Nat. Rev. Gastroenterol. Hepatol. 10, 352–361. 10.1038/nrgastro.2013.35 23478383 PMC3758667

[B122] Kabata H. Moro K. Fukunaga K. Suzuki Y. Miyata J. Masaki K. (2013). Thymic stromal lymphopoietin induces corticosteroid resistance in natural helper cells during airway inflammation. Nat. Commun. 4, 2675. 10.1038/ncomms3675 24157859

[B123] Kahata K. Dadras M. S. Moustakas A. (2018). TGF-Β family signaling in epithelial differentiation and epithelial-mesenchymal transition. Cold Spring Harb. Perspect. Biol. 10, a022194. 10.1101/cshperspect.a022194 28246184 PMC5749157

[B124] Kang C. M. Jang A. S. Ahn M. H. Shin J. A. Kim J. H. Choi Y. S. (2005). Interleukin-25 and interleukin-13 production by alveolar macrophages in response to particles. Am. J. Respir. Cell Mol. Biol. 33, 290–296. 10.1165/rcmb.2005-0003OC 15961726

[B125] Karamanou M. Androutsos G. (2011). Aretaeus of Cappadocia and the first clinical description of asthma. Am. J. Respir. Crit. Care Med. 184, 1420–1421. 10.1164/ajrccm.184.12.1420b 22174116

[B126] Kashyap M. Rochman Y. Spolski R. Samsel L. Leonard W. J. (2011). Thymic stromal lymphopoietin is produced by dendritic cells. J. Immunol. 187, 1207–1211. 10.4049/jimmunol.1100355 21690322 PMC3140600

[B127] Kawamoto A. Nagata S. Anzai S. Takahashi J. Kawai M. Hama M. (2019). Ubiquitin D is upregulated by synergy of Notch signalling and TNF-α in the inflamed intestinal epithelia of IBD patients. J. Crohns Colitis 13, 495–509. 10.1093/ecco-jcc/jjy180 30395194

[B128] Khare V. Krnjic A. Frick A. Gmainer C. Asboth M. Jimenez K. (2019). Mesalamine and azathioprine modulate junctional complexes and restore epithelial barrier function in intestinal inflammation. Sci. Rep. 9, 2842. 10.1038/s41598-019-39401-0 30809073 PMC6391397

[B129] Khounlotham M. Kim W. Peatman E. Nava P. Medina-Contreras O. Addis C. (2012). Compromised intestinal epithelial barrier induces adaptive immune compensation that protects from colitis. Immunity 37, 563–573. 10.1016/j.immuni.2012.06.017 22981539 PMC3564580

[B130] Kierkus J. Dadalski M. Szymanska E. Oracz G. Wegner A. Gorczewska M. (2012). The impact of infliximab induction therapy on mucosal healing and clinical remission in Polish pediatric patients with moderate-to-severe Crohn's disease. Eur. J. Gastroenterol. Hepatol. 24, 495–500. 10.1097/MEG.0b013e32835159f2 22387887

[B131] Kiesslich R. Duckworth C. A. Moussata D. Gloeckner A. Lim L. G. Goetz M. (2012). Local barrier dysfunction identified by confocal laser endomicroscopy predicts relapse in inflammatory bowel disease. Gut 61, 1146–1153. 10.1136/gutjnl-2011-300695 22115910 PMC3388727

[B132] Kiesslich R. Goetz M. Angus E. M. Hu Q. Guan Y. Potten C. (2007). Identification of epithelial gaps in human small and large intestine by confocal endomicroscopy. Gastroenterology 133, 1769–1778. 10.1053/j.gastro.2007.09.011 18054549

[B133] Kinchen J. Chen H. H. Parikh K. Antanaviciute A. Jagielowicz M. Fawkner-Corbett D. (2018). Structural remodeling of the human colonic mesenchyme in inflammatory bowel disease. Cell 175, 372–386. 10.1016/j.cell.2018.08.067 30270042 PMC6176871

[B134] Kips J. C. O'Connor B. J. Inman M. D. Svensson K. Pauwels R. A. O'Byrne P. M. (2000). A long-term study of the antiinflammatory effect of low-dose budesonide plus formoterol versus high-dose budesonide in asthma. Am. J. Respir. Crit. Care Med. 161, 996–1001. 10.1164/ajrccm.161.3.9812056 10712354

[B135] Kirsner J. B. Palmer W. L. (1951). Effect of corticotropin (ACTH) in chronic ulcerative colitis; observations in forty patients. J. Am. Med. Assoc. 147, 541–549. 10.1001/jama.1951.03670230007003 14873501

[B136] Kitson R. R. Moody C. J. (2013). Learning from nature: advances in geldanamycin- and radicicol-based inhibitors of Hsp90. J. Org. Chem. 78, 5117–5141. 10.1021/jo4002849 23496136

[B137] Kohanski M. A. Workman A. D. Patel N. N. Hung L. Y. Shtraks J. P. Chen B. (2018). Solitary chemosensory cells are a primary epithelial source of IL-25 in patients with chronic rhinosinusitis with nasal polyps. J. Allergy Clin. Immunol. 142, 460–469. 10.1016/j.jaci.2018.03.019 29778504 PMC9057652

[B138] Krawiec P. Pac-Kozuchowska E. (2021). Cathelicidin - a novel potential marker of pediatric inflammatory bowel disease. J. Inflamm. Res. 14, 163–174. 10.2147/JIR.S288742 33519224 PMC7837565

[B139] Krndija D. El Marjou F. Guirao B. Richon S. Leroy O. Bellaiche Y. (2019). Active cell migration is critical for steady-state epithelial turnover in the gut. Science 365, 705–710. 10.1126/science.aau3429 31416964

[B140] Krug S. M. Amasheh S. Richter J. F. Milatz S. Gunzel D. Westphal J. K. (2009). Tricellulin forms a barrier to macromolecules in tricellular tight junctions without affecting ion permeability. Mol. Biol. Cell 20, 3713–3724. 10.1091/mbc.e09-01-0080 19535456 PMC2777931

[B141] Krug S. M. Bojarski C. Fromm A. Lee I. M. Dames P. Richter J. F. (2018). Tricellulin is regulated via interleukin-13-receptor α2, affects macromolecule uptake, and is decreased in ulcerative colitis. Mucosal Immunol. 11, 345–356. 10.1038/mi.2017.52 28612843 PMC5730503

[B142] Kuo W. T. Shen L. Zuo L. Shashikanth N. Ong M. Wu L. (2019). Inflammation-induced occludin downregulation limits epithelial apoptosis by suppressing caspase-3 expression. Gastroenterology 157, 1323–1337. 10.1053/j.gastro.2019.07.058 31401143 PMC6815722

[B143] Lambrecht B. N. Hammad H. (2012). The airway epithelium in asthma. Nat. Med. 18, 684–692. 10.1038/nm.2737 22561832

[B144] Lam M. Lamanna E. Organ L. Donovan C. Bourke J. E. (2023). Perspectives on precision cut lung slices-powerful tools for investigation of mechanisms and therapeutic targets in lung diseases. Front. Pharmacol. 14, 1162889. 10.3389/fphar.2023.1162889 37261291 PMC10228656

[B145] Leber A. Hontecillas R. Tubau-Juni N. Zoccoli-Rodriguez V. Abedi V. Bassaganya-Riera J. (2018). NLRX1 modulates immunometabolic mechanisms controlling the host-gut microbiota interactions during inflammatory bowel disease. Front. Immunol. 9, 363. 10.3389/fimmu.2018.00363 29535731 PMC5834749

[B146] Lefrancais E. Duval A. Mirey E. Roga S. Espinosa E. Cayrol C. (2014). Central domain of IL-33 is cleaved by mast cell proteases for potent activation of group-2 innate lymphoid cells. Proc. Natl. Acad. Sci. U. S. A. 111, 15502–15507. 10.1073/pnas.1410700111 25313073 PMC4217470

[B147] Li C. Zhou Y. Rychahou P. Weiss H. L. Lee E. Y. Perry C. L. (2020a). SIRT2 contributes to the regulation of intestinal cell proliferation and differentiation. Cell Mol. Gastroenterol. Hepatol. 10, 43–57. 10.1016/j.jcmgh.2020.01.004 31954883 PMC7210478

[B148] Lichtenstein G. R. Ramsey D. Rubin D. T. (2011). Randomised clinical trial: delayed-release oral mesalazine 4.8 g/day vs. 2.4 g/day in endoscopic mucosal healing--ASCEND I and II combined analysis. Aliment. Pharmacol. Ther. 33, 672–678. 10.1111/j.1365-2036.2010.04575.x 21255059

[B149] Lieleg O. Ribbeck K. (2011). Biological hydrogels as selective diffusion barriers. Trends Cell Biol. 21, 543–551. 10.1016/j.tcb.2011.06.002 21727007 PMC3164742

[B150] Lie M. Van Der Giessen J. Fuhler G. M. De Lima A. Peppelenbosch M. P. Van Der Ent C. (2018). Low dose Naltrexone for induction of remission in inflammatory bowel disease patients. J. Transl. Med. 16, 55. 10.1186/s12967-018-1427-5 29523156 PMC5845217

[B151] Li J. Wang Z. Chu Q. Jiang K. Li J. Tang N. (2018). The strength of mechanical forces determines the differentiation of alveolar epithelial cells. Dev. Cell 44, 297–312. 10.1016/j.devcel.2018.01.008 29408236

[B152] Lim L. G. Neumann J. Hansen T. Goetz M. Hoffman A. Neurath M. F. (2014). Confocal endomicroscopy identifies loss of local barrier function in the duodenum of patients with Crohn's disease and ulcerative colitis. Inflamm. Bowel Dis. 20, 892–900. 10.1097/MIB.0000000000000027 24691113

[B153] Lin W. W. Tsay A. J. Lalime E. N. Pekosz A. Griffin D. E. (2021). Primary differentiated respiratory epithelial cells respond to apical measles virus infection by shedding multinucleated giant cells. Proc. Natl. Acad. Sci. U. S. A. 118, e2013264118. 10.1073/pnas.2013264118 33836570 PMC7980467

[B154] Lin C. Yao E. Zhang K. Jiang X. Croll S. Thompson-Peer K. (2017). YAP is essential for mechanical force production and epithelial cell proliferation during lung branching morphogenesis. Elife 6, e21130. 10.7554/eLife.21130 28323616 PMC5360446

[B155] Liu G. Saren L. Douglasson H. Zhou X. H. Aberg P. M. Ollerstam A. (2021). Precision cut lung slices: an *ex vivo* model for assessing the impact of immunomodulatory therapeutics on lung immune responses. Arch. Toxicol. 95, 2871–2877. 10.1007/s00204-021-03096-y 34191076

[B156] Liu S. Verma M. Michalec L. Liu W. Sripada A. Rollins D. (2018). Steroid resistance of airway type 2 innate lymphoid cells from patients with severe asthma: the role of thymic stromal lymphopoietin. J. Allergy Clin. Immunol. 141, 257–268. 10.1016/j.jaci.2017.03.032 28433687 PMC5650571

[B157] Liu Z. Wu H. Jiang K. Wang Y. Zhang W. Chu Q. (2016). MAPK-mediated YAP activation controls mechanical-tension-induced pulmonary alveolar regeneration. Cell Rep. 16, 1810–1819. 10.1016/j.celrep.2016.07.020 27498861

[B158] Li Y. Zhang T. Guo C. Geng M. Gai S. Qi W. (2020b). Bacillus subtilis RZ001 improves intestinal integrity and alleviates colitis by inhibiting the Notch signalling pathway and activating ATOH-1. Pathog. Dis. 78, ftaa016. 10.1093/femspd/ftaa016 32166323

[B159] Lopez-Posadas R. Becker C. Gunther C. Tenzer S. Amann K. Billmeier U. (2016). Rho-A prenylation and signaling link epithelial homeostasis to intestinal inflammation. J. Clin. Invest. 126, 611–626. 10.1172/JCI80997 26752649 PMC4731169

[B160] Luthi A. U. Cullen S. P. Mcneela E. A. Duriez P. J. Afonina I. S. Sheridan C. (2009). Suppression of interleukin-33 bioactivity through proteolysis by apoptotic caspases. Immunity 31, 84–98. 10.1016/j.immuni.2009.05.007 19559631

[B161] Ma T. Y. Iwamoto G. K. Hoa N. T. Akotia V. Pedram A. Boivin M. A. (2004). TNF-alpha-induced increase in intestinal epithelial tight junction permeability requires NF-kappa B activation. Am. J. Physiol. Gastrointest. Liver Physiol. 286, G367–G376. 10.1152/ajpgi.00173.2003 14766535

[B162] Madara J. L. Stafford J. (1989). Interferon-gamma directly affects barrier function of cultured intestinal epithelial monolayers. J. Clin. Invest. 83, 724–727. 10.1172/JCI113938 2492310 PMC303735

[B163] Madsen K. L. Malfair D. Gray D. Doyle J. S. Jewell L. D. Fedorak R. N. (1999). Interleukin-10 gene-deficient mice develop a primary intestinal permeability defect in response to enteric microflora. Inflamm. Bowel Dis. 5, 262–270. 10.1097/00054725-199911000-00004 10579119

[B164] Mahapatro M. Erkert L. Becker C. (2021). Cytokine-mediated crosstalk between immune cells and epithelial cells in the gut. Cells 10, 111. 10.3390/cells10010111 33435303 PMC7827439

[B165] Mahoney J. E. Mori M. Szymaniak A. D. Varelas X. Cardoso W. V. (2014). The hippo pathway effector Yap controls patterning and differentiation of airway epithelial progenitors. Dev. Cell 30, 137–150. 10.1016/j.devcel.2014.06.003 25043473 PMC6331061

[B166] Marchiando A. M. Shen L. Graham W. V. Weber C. R. Schwarz B. T. Austin J. R. (2010). Caveolin-1-dependent occludin endocytosis is required for TNF-induced tight junction regulation *in vivo* . J. Cell Biol. 189, 111–126. 10.1083/jcb.200902153 20351069 PMC2854371

[B167] Marguet C. Jouen-Boedes F. Dean T. P. Warner J. O. (1999). Bronchoalveolar cell profiles in children with asthma, infantile wheeze, chronic cough, or cystic fibrosis. Am. J. Respir. Crit. Care Med. 159, 1533–1540. 10.1164/ajrccm.159.5.9805028 10228122

[B168] Martens E. C. Roth R. Heuser J. E. Gordon J. I. (2009). Coordinate regulation of glycan degradation and polysaccharide capsule biosynthesis by a prominent human gut symbiont. J. Biol. Chem. 284, 18445–18457. 10.1074/jbc.M109.008094 19403529 PMC2709373

[B169] Martin C. Uhlig S. Ullrich V. (1996). Videomicroscopy of methacholine-induced contraction of individual airways in precision-cut lung slices. Eur. Respir. J. 9, 2479–2487. 10.1183/09031936.96.09122479 8980957

[B170] Martinez-Sanchez L. D. C. Ngo P. A. Pradhan R. Becker L. S. Boehringer D. Soteriou D. (2022). Epithelial RAC1-dependent cytoskeleton dynamics controls cell mechanics, cell shedding and barrier integrity in intestinal inflammation. Gut 72, 275–294. 10.1136/gutjnl-2021-325520 35241625 PMC9872254

[B171] Martinez-Silgado A. Beumer J. Clevers H. (2023). Directed differentiation of murine and human small intestinal organoids toward all mature lineages. Methods Mol. Biol. 2650, 107–122. 10.1007/978-1-0716-3076-1_9 37310627

[B172] Martini E. Krug S. M. Siegmund B. Neurath M. F. Becker C. (2017). Mend your fences: the epithelial barrier and its relationship with mucosal immunity in inflammatory bowel disease. Cell Mol. Gastroenterol. Hepatol. 4, 33–46. 10.1016/j.jcmgh.2017.03.007 28560287 PMC5439240

[B173] Ma Y. Yue J. Zhang Y. Shi C. Odenwald M. Liang W. G. (2017). ACF7 regulates inflammatory colitis and intestinal wound response by orchestrating tight junction dynamics. Nat. Commun. 8, 15375. 10.1038/ncomms15375 28541346 PMC5458510

[B174] Mcclatchey A. I. Yap A. S. (2012). Contact inhibition (of proliferation) redux. Curr. Opin. Cell Biol. 24, 685–694. 10.1016/j.ceb.2012.06.009 22835462

[B175] Mckinley E. T. Sui Y. Al-Kofahi Y. Millis B. A. Tyska M. J. Roland J. T. (2017). Optimized multiplex immunofluorescence single-cell analysis reveals tuft cell heterogeneity. JCI Insight 2, e93487. 10.1172/jci.insight.93487 28570279 PMC5453701

[B176] Meddings J. B. Gibbons I. (1998). Discrimination of site-specific alterations in gastrointestinal permeability in the rat. Gastroenterology 114, 83–92. 10.1016/s0016-5085(98)70636-5 9428222

[B177] Meldrum O. W. Chotirmall S. H. (2021). Mucus, microbiomes and pulmonary disease. Biomedicines 9, 675. 10.3390/biomedicines9060675 34199312 PMC8232003

[B178] Melrose A. G. (1955). The geographical incidence of chronic ulcerative colitis in Britain. Gastroenterology 29, 1055–1060. 10.1016/s0016-5085(19)35927-x 13270830

[B179] Meng X. M. Nikolic-Paterson D. J. Lan H. Y. (2016). TGF-β: the master regulator of fibrosis. Nat. Rev. Nephrol. 12, 325–338. 10.1038/nrneph.2016.48 27108839

[B180] Moheimani F. Hsu A. C. Reid A. T. Williams T. Kicic A. Stick S. M. (2016). The genetic and epigenetic landscapes of the epithelium in asthma. Respir. Res. 17, 119. 10.1186/s12931-016-0434-4 27658857 PMC5034566

[B181] Montassier E. Kitsios G. D. Radder J. E. Le Bastard Q. Kelly B. J. Panzer A. (2023). Robust airway microbiome signatures in acute respiratory failure and hospital-acquired pneumonia. Nat. Med. 29, 2793–2804. 10.1038/s41591-023-02617-9 37957375

[B182] Montoro D. T. Haber A. L. Biton M. Vinarsky V. Lin B. Birket S. E. (2018). A revised airway epithelial hierarchy includes CFTR-expressing ionocytes. Nature 560, 319–324. 10.1038/s41586-018-0393-7 30069044 PMC6295155

[B183] Mookherjee N. Anderson M. A. Haagsman H. P. Davidson D. J. (2020). Antimicrobial host defence peptides: functions and clinical potential. Nat. Rev. Drug Discov. 19, 311–332. 10.1038/s41573-019-0058-8 32107480

[B184] Moor A. E. Harnik Y. Ben-Moshe S. Massasa E. E. Rozenberg M. Eilam R. (2018). Spatial reconstruction of single enterocytes uncovers broad zonation along the intestinal villus Axis. Cell 175, 1156–1167. 10.1016/j.cell.2018.08.063 30270040

[B185] Moshiri J. Craven A. R. Mixon S. B. Amieva M. R. Kirkegaard K. (2023). Mechanosensitive extrusion of Enterovirus A71-infected cells from colonic organoids. Nat. Microbiol. 8, 629–639. 10.1038/s41564-023-01339-5 36914754 PMC10066035

[B186] Munkholm P. Langholz E. Hollander D. Thornberg K. Orholm M. Katz K. D. (1994). Intestinal permeability in patients with Crohn's disease and ulcerative colitis and their first degree relatives. Gut 35, 68–72. 10.1136/gut.35.1.68 8307453 PMC1374635

[B187] Naydenov N. G. Feygin A. Wang D. Kuemmerle J. F. Harris G. Conti M. A. (2016). Nonmuscle myosin IIA regulates intestinal epithelial barrier *in vivo* and plays a protective role during experimental colitis. Sci. Rep. 6, 24161. 10.1038/srep24161 27063635 PMC4827066

[B188] Neurath M. F. Travis S. P. (2012). Mucosal healing in inflammatory bowel diseases: a systematic review. Gut 61, 1619–1635. 10.1136/gutjnl-2012-302830 22842618

[B189] Neurath M. F. Vieth M. (2023). Different levels of healing in inflammatory bowel diseases: mucosal, histological, transmural, barrier and complete healing. Gut 72, 2164–2183. 10.1136/gutjnl-2023-329964 37640443

[B190] Nowarski R. Jackson R. Gagliani N. De Zoete M. R. Palm N. W. Bailis W. (2015). Epithelial IL-18 equilibrium controls barrier function in colitis. Cell 163, 1444–1456. 10.1016/j.cell.2015.10.072 26638073 PMC4943028

[B191] Olson T. S. Reuter B. K. Scott K. G. Morris M. A. Wang X. M. Hancock L. N. (2006). The primary defect in experimental ileitis originates from a nonhematopoietic source. J. Exp. Med. 203, 541–552. 10.1084/jem.20050407 16505137 PMC2118253

[B192] Ortiz-Zapater E. Bagley D. C. Hernandez V. L. Roberts L. B. Maguire T. J. A. Voss F. (2022a). Epithelial coxsackievirus adenovirus receptor promotes house dust mite-induced lung inflammation. Nat. Commun. 13, 6407. 10.1038/s41467-022-33882-w 36302767 PMC9613683

[B193] Ortiz-Zapater E. Signes-Costa J. Montero P. Roger I. (2022b). Lung fibrosis and fibrosis in the lungs: is it all about myofibroblasts? Biomedicines 10, 1423. 10.3390/biomedicines10061423 35740444 PMC9220162

[B194] Oshima T. Miwa H. Joh T. (2008). Changes in the expression of claudins in active ulcerative colitis. J. Gastroenterol. Hepatol. 23 (Suppl. 2), S146–S150. 10.1111/j.1440-1746.2008.05405.x 19120888

[B195] Palm N. W. De Zoete M. R. Cullen T. W. Barry N. A. Stefanowski J. Hao L. (2014). Immunoglobulin A coating identifies colitogenic bacteria in inflammatory bowel disease. Cell 158, 1000–1010. 10.1016/j.cell.2014.08.006 25171403 PMC4174347

[B196] Panek M. Stawiski K. Kaszkowiak M. Kuna P. (2022). Cytokine TGFβ gene polymorphism in asthma: TGF-related SNP analysis enhances the prediction of disease diagnosis (A case-control study with multivariable data-mining model development). Front. Immunol. 13, 746360. 10.3389/fimmu.2022.746360 35774789 PMC9238410

[B197] Pannekoek W. J. De Rooij J. Gloerich M. (2019). Force transduction by cadherin adhesions in morphogenesis. F1000Res 8, F1000 Faculty Rev-1044. 10.12688/f1000research.18779.1 PMC6625547 31327995

[B198] Paone P. Cani P. D. (2020). Mucus barrier, mucins and gut microbiota: the expected slimy partners? Gut 69, 2232–2243. 10.1136/gutjnl-2020-322260 32917747 PMC7677487

[B199] Papi A. Brightling C. Pedersen S. E. Reddel H. K. (2018). Asthma. Lancet 391, 783–800. 10.1016/S0140-6736(17)33311-1 29273246

[B200] Parikh K. Antanaviciute A. Fawkner-Corbett D. Jagielowicz M. Aulicino A. Lagerholm C. (2019). Colonic epithelial cell diversity in health and inflammatory bowel disease. Nature 567, 49–55. 10.1038/s41586-019-0992-y 30814735

[B201] Park J. A. Fredberg J. J. Drazen J. M. (2015). Putting the squeeze on airway epithelia. Physiol. (Bethesda) 30, 293–303. 10.1152/physiol.00004.2015 PMC4888965 26136543

[B202] Park J. A. Tschumperlin D. J. (2009). Chronic intermittent mechanical stress increases MUC5AC protein expression. Am. J. Respir. Cell Mol. Biol. 41, 459–466. 10.1165/rcmb.2008-0195OC 19168703 PMC2746990

[B203] Pavord I. D. Beasley R. Agusti A. Anderson G. P. Bel E. Brusselle G. (2018). After asthma: redefining airways diseases. Lancet 391, 350–400. 10.1016/S0140-6736(17)30879-6 28911920

[B204] Payne D. N. Rogers A. V. Adelroth E. Bandi V. Guntupalli K. K. Bush A. (2003). Early thickening of the reticular basement membrane in children with difficult asthma. Am. J. Respir. Crit. Care Med. 167, 78–82. 10.1164/rccm.200205-414OC 12502479

[B205] Perez-Gonzalez C. Ceada G. Greco F. Matejcic M. Gomez-Gonzalez M. Castro N. (2021). Mechanical compartmentalization of the intestinal organoid enables crypt folding and collective cell migration. Nat. Cell Biol. 23, 745–757. 10.1038/s41556-021-00699-6 34155382 PMC7611697

[B206] Perez-Gonzalez C. Ceada G. Matejcic M. Trepat X. (2022). Digesting the mechanobiology of the intestinal epithelium. Curr. Opin. Genet. Dev. 72, 82–90. 10.1016/j.gde.2021.10.005 34902705

[B207] Pezzulo A. A. Tudas R. A. Stewart C. G. Buonfiglio L. G. V. Lindsay B. D. Taft P. J. (2019). HSP90 inhibitor geldanamycin reverts IL-13- and IL-17-induced airway goblet cell metaplasia. J. Clin. Invest. 129, 744–758. 10.1172/JCI123524 30640172 PMC6355221

[B208] Pinkerton J. W. Kim R. Y. Koeninger L. Armbruster N. S. Hansbro N. G. Brown A. C. (2021). Human β-defensin-2 suppresses key features of asthma in murine models of allergic airways disease. Clin. Exp. Allergy 51, 120–131. 10.1111/cea.13766 33098152

[B209] Piyadasa H. Hemshekhar M. Altieri A. Basu S. Van Der Does A. M. Halayko A. J. (2018). Immunomodulatory innate defence regulator (IDR) peptide alleviates airway inflammation and hyper-responsiveness. Thorax 73, 908–917. 10.1136/thoraxjnl-2017-210739 29853649

[B210] Plasschaert L. W. Zilionis R. Choo-Wing R. Savova V. Knehr J. Roma G. (2018). A single-cell atlas of the airway epithelium reveals the CFTR-rich pulmonary ionocyte. Nature 560, 377–381. 10.1038/s41586-018-0394-6 30069046 PMC6108322

[B211] Pohunek P. Warner J. O. Turzikova J. Kudrmann J. Roche W. R. (2005). Markers of eosinophilic inflammation and tissue re-modelling in children before clinically diagnosed bronchial asthma. Pediatr. Allergy Immunol. 16, 43–51. 10.1111/j.1399-3038.2005.00239.x 15693911

[B212] Porsbjerg C. Melen E. Lehtimaki L. Shaw D. (2023). Asthma. Lancet 401, 858–873. 10.1016/S0140-6736(22)02125-0 36682372

[B213] Probert C. S. Dignass A. U. Lindgren S. Oudkerk Pool M. Marteau P. (2014). Combined oral and rectal mesalazine for the treatment of mild-to-moderately active ulcerative colitis: rapid symptom resolution and improvements in quality of life. J. Crohns Colitis 8, 200–207. 10.1016/j.crohns.2013.08.007 24012063

[B214] Puliafito A. Hufnagel L. Neveu P. Streichan S. Sigal A. Fygenson D. K. (2012). Collective and single cell behavior in epithelial contact inhibition. Proc. Natl. Acad. Sci. U. S. A. 109, 739–744. 10.1073/pnas.1007809109 22228306 PMC3271933

[B215] Quansah E. Gardey E. Ramoji A. Meyer-Zedler T. Goehrig B. Heutelbeck A. (2023). Intestinal epithelial barrier integrity investigated by label-free techniques in ulcerative colitis patients. Sci. Rep. 13, 2681. 10.1038/s41598-023-29649-y 36792686 PMC9931702

[B216] Raju P. Shashikanth N. Tsai P. Y. Pongkorpsakol P. Chanez-Paredes S. Steinhagen P. R. (2020). Inactivation of paracellular cation-selective claudin-2 channels attenuates immune-mediated experimental colitis in mice. J. Clin. Invest. 130, 5197–5208. 10.1172/JCI138697 32516134 PMC7524482

[B217] Raleigh D. R. Boe D. M. Yu D. Weber C. R. Marchiando A. M. Bradford E. M. (2011). Occludin S408 phosphorylation regulates tight junction protein interactions and barrier function. J. Cell Biol. 193, 565–582. 10.1083/jcb.201010065 21536752 PMC3087007

[B218] Rallabandi H. R. Yang H. Oh K. B. Lee H. C. Byun S. J. Lee B. R. (2020). Evaluation of intestinal epithelial barrier function in inflammatory bowel diseases using murine intestinal organoids. Tissue Eng. Regen. Med. 17, 641–650. 10.1007/s13770-020-00278-0 32594459 PMC7524940

[B220] Ramsey K. A. Chen A. C. H. Radicioni G. Lourie R. Martin M. Broomfield A. (2020). Airway mucus hyperconcentration in non-cystic fibrosis bronchiectasis. Am. J. Respir. Crit. Care Med. 201, 661–670. 10.1164/rccm.201906-1219OC 31765597 PMC7068838

[B221] Rath T. Atreya R. Bodenschatz J. Uter W. Geppert C. E. Vitali F. (2023). Intestinal barrier healing is superior to endoscopic and histologic remission for predicting major adverse outcomes in inflammatory bowel disease: the prospective ERIca trial. Gastroenterology 164, 241–255. 10.1053/j.gastro.2022.10.014 36279923

[B222] Rath T. Atreya R. Neurath M. F. (2021). Is histological healing a feasible endpoint in ulcerative colitis? Expert Rev. Gastroenterol. Hepatol. 15, 665–674. 10.1080/17474124.2021.1880892 33481635

[B223] Regev A. Teichmann S. A. Lander E. S. Amit I. Benoist C. Birney E. (2017). The human cell atlas. Elife 6, e27041. 10.7554/eLife.27041 29206104 PMC5762154

[B224] Ressler B. Lee R. T. Randell S. H. Drazen J. M. Kamm R. D. (2000). Molecular responses of rat tracheal epithelial cells to transmembrane pressure. Am. J. Physiol. Lung Cell Mol. Physiol. 278, L1264–L1272. 10.1152/ajplung.2000.278.6.L1264 10835333

[B225] Resta-Lenert S. Smitham J. Barrett K. E. (2005). Epithelial dysfunction associated with the development of colitis in conventionally housed mdr1a-/- mice. Am. J. Physiol. Gastrointest. Liver Physiol. 289, G153–G162. 10.1152/ajpgi.00395.2004 15774938

[B226] Reynolds A. Wharton N. Parris A. Mitchell E. Sobolewski A. Kam C. (2014). Canonical Wnt signals combined with suppressed TGFβ/BMP pathways promote renewal of the native human colonic epithelium. Gut 63, 610–621. 10.1136/gutjnl-2012-304067 23831735 PMC3963552

[B227] Roan F. Obata-Ninomiya K. Ziegler S. F. (2019). Epithelial cell-derived cytokines: more than just signaling the alarm. J. Clin. Invest. 129, 1441–1451. 10.1172/JCI124606 30932910 PMC6436879

[B228] Roodsant T. Navis M. Aknouch I. Renes I. B. Van Elburg R. M. Pajkrt D. (2020). A human 2D primary organoid-derived epithelial monolayer model to study host-pathogen interaction in the small intestine. Front. Cell Infect. Microbiol. 10, 272. 10.3389/fcimb.2020.00272 32656095 PMC7326037

[B229] Rosales Gerpe M. C. Van Vloten J. P. Santry L. A. De Jong J. Mould R. C. Pelin A. (2018). Use of precision-cut lung slices as an *ex vivo* tool for evaluating viruses and viral vectors for gene and oncolytic therapy. Mol. Ther. Methods Clin. Dev. 10, 245–256. 10.1016/j.omtm.2018.07.010 30112421 PMC6092314

[B230] Rosenblatt J. Raff M. C. Cramer L. P. (2001). An epithelial cell destined for apoptosis signals its neighbors to extrude it by an actin- and myosin-dependent mechanism. Curr. Biol. 11, 1847–1857. 10.1016/s0960-9822(01)00587-5 11728307

[B231] Roy S. Esmaeilniakooshkghazi A. Patnaik S. Wang Y. George S. P. Ahrorov A. (2018). Villin-1 and Gelsolin regulate changes in actin dynamics that affect cell survival signaling pathways and intestinal inflammation. Gastroenterology 154, 1405–1420. 10.1053/j.gastro.2017.12.016 29274870 PMC7808315

[B232] Runge S. Rosshart S. P. (2021). The mammalian metaorganism: a holistic view on how microbes of all kingdoms and niches shape local and systemic immunity. Front. Immunol. 12, 702378. 10.3389/fimmu.2021.702378 34276696 PMC8278200

[B233] Rupani H. Fong W. C. G. Kyyaly A. Kurukulaaratchy R. J. (2021). Recent insights into the management of inflammation in asthma. J. Inflamm. Res. 14, 4371–4397. 10.2147/JIR.S295038 34511973 PMC8421249

[B234] Rutgeerts P. Van Assche G. Sandborn W. J. Wolf D. C. Geboes K. Colombel J. F. (2012). Adalimumab induces and maintains mucosal healing in patients with Crohn's disease: data from the EXTEND trial. Gastroenterology 142, 1102–1111 e2. 10.1053/j.gastro.2012.01.035 22326435

[B235] Saenz S. A. Siracusa M. C. Perrigoue J. G. Spencer S. P. Urban J. F. JR. Tocker J. E. (2010). IL25 elicits a multipotent progenitor cell population that promotes T(H)2 cytokine responses. Nature 464, 1362–1366. 10.1038/nature08901 20200520 PMC2861732

[B236] Saito A. C. Higashi T. Fukazawa Y. Otani T. Tauchi M. Higashi A. Y. (2021). Occludin and tricellulin facilitate formation of anastomosing tight-junction strand network to improve barrier function. Mol. Biol. Cell 32, 722–738. 10.1091/mbc.E20-07-0464 33566640 PMC8108510

[B237] Salimi M. Barlow J. L. Saunders S. P. Xue L. Gutowska-Owsiak D. Wang X. (2013). A role for IL-25 and IL-33-driven type-2 innate lymphoid cells in atopic dermatitis. J. Exp. Med. 210, 2939–2950. 10.1084/jem.20130351 24323357 PMC3865470

[B238] Sanchez-Guzman D. Boland S. Brookes O. Mc Cord C. Lai Kuen R. Sirri V. (2021). Long-term evolution of the epithelial cell secretome in preclinical 3D models of the human bronchial epithelium. Sci. Rep. 11, 6621. 10.1038/s41598-021-86037-0 33758289 PMC7988136

[B239] Sanderson I. R. Boulton P. Menzies I. Walker-Smith J. A. (1987). Improvement of abnormal lactulose/rhamnose permeability in active Crohn's disease of the small bowel by an elemental diet. Gut 28, 1073–1076. 10.1136/gut.28.9.1073 3678965 PMC1433221

[B240] Saxena K. Blutt S. E. Ettayebi K. Zeng X. L. Broughman J. R. Crawford S. E. (2016). Human intestinal enteroids: a new model to study human rotavirus infection, host restriction, and pathophysiology. J. Virol. 90, 43–56. 10.1128/JVI.01930-15 26446608 PMC4702582

[B241] Schaubeck M. Clavel T. Calasan J. Lagkouvardos I. Haange S. B. Jehmlich N. (2016). Dysbiotic gut microbiota causes transmissible Crohn’s disease-like ileitis independent of failure in antimicrobial defence. Gut 65, 225–237. 10.1136/gutjnl-2015-309333 25887379 PMC4752651

[B242] Schleich F. Sabbe M. Moermans C. Louis R. (2024). Tezepelumab (Tezspire®): new biological treatment of severe asthma. Rev. Med. Liege 79, 60–64. 38223972

[B243] Schroeder B. O. (2019). Fight them or feed them: how the intestinal mucus layer manages the gut microbiota. Gastroenterol. Rep. (Oxf) 7, 3–12. 10.1093/gastro/goy052 30792861 PMC6375348

[B244] Schwarz B. T. Wang F. Shen L. Clayburgh D. R. Su L. Wang Y. (2007). LIGHT signals directly to intestinal epithelia to cause barrier dysfunction via cytoskeletal and endocytic mechanisms. Gastroenterology 132, 2383–2394. 10.1053/j.gastro.2007.02.052 17570213 PMC2709832

[B245] Seidelin J. B. Bjerrum J. T. Coskun M. Widjaya B. Vainer B. Nielsen O. H. (2010). IL-33 is upregulated in colonocytes of ulcerative colitis. Immunol. Lett. 128, 80–85. 10.1016/j.imlet.2009.11.001 19913053

[B246] Shahana S. Bjornsson E. Ludviksdottir D. Janson C. Nettelbladt O. Venge P. (2005). Ultrastructure of bronchial biopsies from patients with allergic and non-allergic asthma. Respir. Med. 99, 429–443. 10.1016/j.rmed.2004.08.013 15763449

[B247] Sheih A. Parks W. C. Ziegler S. F. (2017). GM-CSF produced by the airway epithelium is required for sensitization to cockroach allergen. Mucosal Immunol. 10, 705–715. 10.1038/mi.2016.90 27731325 PMC5389932

[B248] Shin W. Kim H. J. (2022). 3D *in vitro* morphogenesis of human intestinal epithelium in a gut-on-a-chip or a hybrid chip with a cell culture insert. Nat. Protoc. 17, 910–939. 10.1038/s41596-021-00674-3 35110737 PMC9675318

[B249] Simms L. A. Doecke J. D. Walsh M. D. Huang N. Fowler E. V. Radford-Smith G. L. (2008). Reduced alpha-defensin expression is associated with inflammation and not NOD2 mutation status in ileal Crohn’s disease. Gut 57, 903–910. 10.1136/gut.2007.142588 18305068

[B250] Sivagnanam M. Mueller J. L. Lee H. Chen Z. Nelson S. F. Turner D. (2008). Identification of EpCAM as the gene for congenital tufting enteropathy. Gastroenterology 135, 429–437. 10.1053/j.gastro.2008.05.036 18572020 PMC2574708

[B251] Soderholm J. D. Olaison G. Lindberg E. Hannestad U. Vindels A. Tysk C. (1999). Different intestinal permeability patterns in relatives and spouses of patients with Crohn's disease: an inherited defect in mucosal defence? Gut 44, 96–100. 10.1136/gut.44.1.96 9862833 PMC1760070

[B252] Song C. Chai Z. Chen S. Zhang H. Zhang X. Zhou Y. (2023). Intestinal mucus components and secretion mechanisms: what we do and do not know. Exp. Mol. Med. 55, 681–691. 10.1038/s12276-023-00960-y 37009791 PMC10167328

[B253] Stock P. Lombardi V. Kohlrautz V. Akbari O. (2009). Induction of airway hyperreactivity by IL-25 is dependent on a subset of invariant NKT cells expressing IL-17RB. J. Immunol. 182, 5116–5122. 10.4049/jimmunol.0804213 19342692 PMC2837931

[B254] Streichan S. J. Hoerner C. R. Schneidt T. Holzer D. Hufnagel L. (2014). Spatial constraints control cell proliferation in tissues. Proc. Natl. Acad. Sci. U. S. A. 111, 5586–5591. 10.1073/pnas.1323016111 24706777 PMC3992650

[B255] Sturgeon C. Lan J. Fasano A. (2017). Zonulin transgenic mice show altered gut permeability and increased morbidity/mortality in the DSS colitis model. Ann. N. Y. Acad. Sci. 1397, 130–142. 10.1111/nyas.13343 28423466 PMC5479715

[B256] Suenaert P. Bulteel V. Lemmens L. Noman M. Geypens B. Van Assche G. (2002). Anti-tumor necrosis factor treatment restores the gut barrier in Crohn's disease. Am. J. Gastroenterol. 97, 2000–2004. 10.1111/j.1572-0241.2002.05914.x 12190167

[B257] Sugawara T. Furuse K. Otani T. Wakayama T. Furuse M. (2021). Angulin-1 seals tricellular contacts independently of tricellulin and claudins. J. Cell Biol. 220, e202005062. 10.1083/jcb.202005062 34269802 PMC8289698

[B258] Su L. Nalle S. C. Shen L. Turner E. S. Singh G. Breskin L. A. (2013). TNFR2 activates MLCK-dependent tight junction dysregulation to cause apoptosis-mediated barrier loss and experimental colitis. Gastroenterology 145, 407–415. 10.1053/j.gastro.2013.04.011 23619146 PMC3722284

[B259] Sumigray K. D. Terwilliger M. Lechler T. (2018). Morphogenesis and compartmentalization of the intestinal crypt. Dev. Cell 45, 183–197. 10.1016/j.devcel.2018.03.024 29689194 PMC5987226

[B260] Suzuki T. Yoshinaga N. Tanabe S. (2011). Interleukin-6 (IL-6) regulates claudin-2 expression and tight junction permeability in intestinal epithelium. J. Biol. Chem. 286, 31263–31271. 10.1074/jbc.M111.238147 21771795 PMC3173073

[B261] Tahaghoghi-Hajghorbani S. Ajami A. Ghorbanalipoor S. Hosseini-Khah Z. Taghiloo S. Khaje-Enayati P. (2019). Protective effect of TSLP and IL-33 cytokines in ulcerative colitis. Auto. Immun. Highlights 10, 1. 10.1186/s13317-019-0110-z 30868311 PMC6416230

[B262] Takeyama K. Kondo M. Akaba T. Tamaoki J. (2015). Profile of airway mucins in bronchoalveolar lavage fluid of patients with pulmonary alveolar proteinosis. (European Respiratory Journal, 46, PA3870. 10.1183/13993003.congress-2015.PA3870

[B263] Tan H. T. Hagner S. Ruchti F. Radzikowska U. Tan G. Altunbulakli C. (2019). Tight junction, mucin, and inflammasome-related molecules are differentially expressed in eosinophilic, mixed, and neutrophilic experimental asthma in mice. Allergy 74, 294–307. 10.1111/all.13619 30267575

[B264] Tang W. Li M. Teng F. Cui J. Dong J. Wang W. (2022). Single-cell RNA-sequencing in asthma research. Front. Immunol. 13, 988573. 10.3389/fimmu.2022.988573 36524132 PMC9744750

[B265] Taylor B. C. Zaph C. Troy A. E. Du Y. Guild K. J. Comeau M. R. (2009). TSLP regulates intestinal immunity and inflammation in mouse models of helminth infection and colitis. J. Exp. Med. 206, 655–667. 10.1084/jem.20081499 19273626 PMC2699121

[B266] Teshima C. W. Dieleman L. A. Meddings J. B. (2012). Abnormal intestinal permeability in Crohn's disease pathogenesis. Ann. N. Y. Acad. Sci. 1258, 159–165. 10.1111/j.1749-6632.2012.06612.x 22731729

[B267] Teshima C. W. Meddings J. B. (2008). The measurement and clinical significance of intestinal permeability. Curr. Gastroenterol. Rep. 10, 443–449. 10.1007/s11894-008-0083-y 18799118

[B268] Thornton D. J. Rousseau K. Mcguckin M. A. (2008). Structure and function of the polymeric mucins in airways mucus. Annu. Rev. Physiol. 70, 459–486. 10.1146/annurev.physiol.70.113006.100702 17850213

[B269] Tibble J. A. Sigthorsson G. Bridger S. Fagerhol M. K. Bjarnason I. (2000). Surrogate markers of intestinal inflammation are predictive of relapse in patients with inflammatory bowel disease. Gastroenterology 119, 15–22. 10.1053/gast.2000.8523 10889150

[B270] Topczewska P. M. Rompe Z. A. Jakob M. O. Stamm A. Leclère P. S. Preusser A. (2023). ILC2 require cell-intrinsic ST2 signals to promote type 2 immune responses. Front. Immunol. 14, 1130933. 10.3389/fimmu.2023.1130933 37063913 PMC10104602

[B271] Travaglini K. J. Nabhan A. N. Penland L. Sinha R. Gillich A. Sit R. V. (2020). A molecular cell atlas of the human lung from single-cell RNA sequencing. Nature 587, 619–625. 10.1038/s41586-020-2922-4 33208946 PMC7704697

[B272] Travers J. Rochman M. Miracle C. E. Habel J. E. Brusilovsky M. Caldwell J. M. (2018). Chromatin regulates IL-33 release and extracellular cytokine activity. Nat. Commun. 9, 3244. 10.1038/s41467-018-05485-x 30108214 PMC6092330

[B273] Treveil A. Sudhakar P. Matthews Z. J. Wrzesinski T. Jones E. J. Brooks J. (2020). Regulatory network analysis of Paneth cell and goblet cell enriched gut organoids using transcriptomics approaches. Mol. Omics 16, 39–58. 10.1039/c9mo00130a 31819932

[B274] Tschumperlin D. J. Shively J. D. Kikuchi T. Drazen J. M. (2003). Mechanical stress triggers selective release of fibrotic mediators from bronchial epithelium. Am. J. Respir. Cell Mol. Biol. 28, 142–149. 10.1165/rcmb.2002-0121OC 12540481

[B275] Turpin W. Lee S. H. Raygoza Garay J. A. Madsen K. L. Meddings J. B. Bedrani L. (2020). Increased intestinal permeability is associated with later development of crohn's disease. Gastroenterology 159, 2092–2100 e5. 10.1053/j.gastro.2020.08.005 32791132

[B276] Tytgat K. M. Van Der Wal J. W. Einerhand A. W. Buller H. A. Dekker J. (1996). Quantitative analysis of MUC2 synthesis in ulcerative colitis. Biochem. Biophys. Res. Commun. 224, 397–405. 10.1006/bbrc.1996.1039 8702401

[B277] Uroz M. Wistorf S. Serra-Picamal X. Conte V. Sales-Pardo M. Roca-Cusachs P. (2018). Regulation of cell cycle progression by cell-cell and cell-matrix forces. Nat. Cell Biol. 20, 646–654. 10.1038/s41556-018-0107-2 29802405

[B278] Van Der Lugt B. Van Beek A. A. Aalvink S. Meijer B. Sovran B. Vermeij W. P. (2019). Akkermansia muciniphila ameliorates the age-related decline in colonic mucus thickness and attenuates immune activation in accelerated aging Ercc1 -/Δ7 mice. Immun. Ageing 16, 6. 10.1186/s12979-019-0145-z 30899315 PMC6408808

[B279] Van De Wetering M. Francies H. E. Francis J. M. Bounova G. Iorio F. Pronk A. (2015). Prospective derivation of a living organoid biobank of colorectal cancer patients. Cell 161, 933–945. 10.1016/j.cell.2015.03.053 25957691 PMC6428276

[B280] Van Itallie C. M. Fanning A. S. Holmes J. Anderson J. M. (2010). Occludin is required for cytokine-induced regulation of tight junction barriers. J. Cell Sci. 123, 2844–2852. 10.1242/jcs.065581 20663912 PMC2915885

[B281] Van Rijt L. S. Vos N. Willart M. Muskens F. Tak P. P. Van Der Horst C. (2011). Persistent activation of dendritic cells after resolution of allergic airway inflammation breaks tolerance to inhaled allergens in mice. Am. J. Respir. Crit. Care Med. 184, 303–311. 10.1164/rccm.201101-0019OC 21562124

[B282] Vasanthakumar A. Moro K. Xin A. Liao Y. Gloury R. Kawamoto S. (2015). The transcriptional regulators IRF4, BATF and IL-33 orchestrate development and maintenance of adipose tissue-resident regulatory T cells. Nat. Immunol. 16, 276–285. 10.1038/ni.3085 25599561

[B283] Veerati P. C. Mitchel J. A. Reid A. T. Knight D. A. Bartlett N. W. Park J. A. (2020). Airway mechanical compression: its role in asthma pathogenesis and progression. Eur. Respir. Rev. 29, 190123. 10.1183/16000617.0123-2019 32759373 PMC8008491

[B284] Vieira Braga F. A. Kar G. Berg M. Carpaij O. A. Polanski K. Simon L. M. (2019). A cellular census of human lungs identifies novel cell states in health and in asthma. Nat. Med. 25, 1153–1163. 10.1038/s41591-019-0468-5 31209336

[B285] Vivinus-Nebot M. Frin-Mathy G. Bzioueche H. Dainese R. Bernard G. Anty R. (2014). Functional bowel symptoms in quiescent inflammatory bowel diseases: role of epithelial barrier disruption and low-grade inflammation. Gut 63, 744–752. 10.1136/gutjnl-2012-304066 23878165

[B286] Von Moltke J. Ji M. Liang H. E. Locksley R. M. (2016). Tuft-cell-derived IL-25 regulates an intestinal ILC2-epithelial response circuit. Nature 529, 221–225. 10.1038/nature16161 26675736 PMC4830391

[B287] Wang F. Graham W. V. Wang Y. Witkowski E. D. Schwarz B. T. Turner J. R. (2005). Interferon-gamma and tumor necrosis factor-alpha synergize to induce intestinal epithelial barrier dysfunction by up-regulating myosin light chain kinase expression. Am. J. Pathol. 166, 409–419. 10.1016/s0002-9440(10)62264-x 15681825 PMC1237049

[B288] Warren S. Sommers S. C. (1948). Cicatrizing enteritis as a pathologic entity; analysis of 120 cases. Am. J. Pathol. 24, 475–501. 18859355 PMC1942793

[B289] Watson A. J. M. Duckworth C. A. Guan Y. Montrose M. H. (2009). Mechanisms of epithelial cell shedding in the Mammalian intestine and maintenance of barrier function. Ann. N. Y. Acad. Sci. 1165, 135–142. 10.1111/j.1749-6632.2009.04027.x 19538298

[B290] Wawrzyniak P. Wawrzyniak M. Wanke K. Sokolowska M. Bendelja K. Ruckert B. (2017). Regulation of bronchial epithelial barrier integrity by type 2 cytokines and histone deacetylases in asthmatic patients. J. Allergy Clin. Immunol. 139, 93–103. 10.1016/j.jaci.2016.03.050 27312821

[B291] Weber C. R. Raleigh D. R. Su L. Shen L. Sullivan E. A. Wang Y. (2010). Epithelial myosin light chain kinase activation induces mucosal interleukin-13 expression to alter tight junction ion selectivity. J. Biol. Chem. 285, 12037–12046. 10.1074/jbc.M109.064808 20177070 PMC2852941

[B292] Weber F. (2014). Antiviral innate immunity: introduction☆. *Reference Module in biomedical sciences* . Elsevier. 2014 cited 2023 May 31. 10.1016/B978-0-12-801238-3.01886-9

[B293] Wehkamp J. Salzman N. H. Porter E. Nuding S. Weichenthal M. Petras R. E. (2005). Reduced Paneth cell alpha-defensins in ileal Crohn’s disease. Proc. Natl. Acad. Sci. U. S. A. 102, 18129–18134. 10.1073/pnas.0505256102 16330776 PMC1306791

[B294] Welsh K. G. Rousseau K. Fisher G. Bonser L. R. Bradding P. Brightling C. E. (2017). MUC5AC and a glycosylated variant of MUC5B alter mucin composition in children with acute asthma. Chest 152, 771–779. 10.1016/j.chest.2017.07.001 28716644 PMC5624091

[B295] Widdicombe J. H. (2019). Early studies on the surface epithelium of mammalian airways. Am. J. Physiol. Lung Cell Mol. Physiol. 317, L486–L495. 10.1152/ajplung.00240.2019 31313615

[B296] Wild G. E. Waschke K. A. Bitton A. Thomson A. B. (2003). The mechanisms of prednisone inhibition of inflammation in Crohn's disease involve changes in intestinal permeability, mucosal TNFalpha production and nuclear factor kappa B expression. Aliment. Pharmacol. Ther. 18, 309–317. 10.1046/j.1365-2036.2003.01611.x 12895215

[B297] Wlodarska M. Luo C. Kolde R. D'Hennezel E. Annand J. W. Heim C. E. (2017). Indoleacrylic acid produced by commensal peptostreptococcus species suppresses inflammation. Cell Host Microbe 22, 25–37. 10.1016/j.chom.2017.06.007 28704649 PMC5672633

[B298] Wu W. Lv L. Shi D. Ye J. Fang D. Guo F. (2017). Protective effect of akkermansia muciniphila against immune-mediated liver injury in a mouse model. Front. Microbiol. 8, 1804. 10.3389/fmicb.2017.01804 29033903 PMC5626943

[B299] Wyatt J. Vogelsang H. Hubl W. Waldhoer T. Lochs H. (1993). Intestinal permeability and the prediction of relapse in Crohn's disease. Lancet 341, 1437–1439. 10.1016/0140-6736(93)90882-h 8099141

[B300] Xu P. Elizalde M. Masclee A. Pierik M. Jonkers D. (2021). Corticosteroid enhances epithelial barrier function in intestinal organoids derived from patients with Crohn's disease. J. Mol. Med. Berl. 99, 805–815. 10.1007/s00109-021-02045-7 33575854 PMC8164603

[B301] Yang Q. Xue S. L. Chan C. J. Rempfler M. Vischi D. Maurer-Gutierrez F. (2021). Cell fate coordinates mechano-osmotic forces in intestinal crypt formation. Nat. Cell Biol. 23, 733–744. 10.1038/s41556-021-00700-2 34155381 PMC7611267

[B302] Yoo J. H. Donowitz M. (2019). Intestinal enteroids/organoids: a novel platform for drug discovery in inflammatory bowel diseases. World J. Gastroenterol. 25, 4125–4147. 10.3748/wjg.v25.i30.4125 31435168 PMC6700704

[B303] Yui S. Azzolin L. Maimets M. Pedersen M. T. Fordham R. P. Hansen S. L. (2018). YAP/TAZ-Dependent reprogramming of colonic epithelium links ECM remodeling to tissue regeneration. Cell Stem Cell 22, 35–49. 10.1016/j.stem.2017.11.001 29249464 PMC5766831

[B304] Yui S. Nakamura T. Sato T. Nemoto Y. Mizutani T. Zheng X. (2012). Functional engraftment of colon epithelium expanded *in vitro* from a single adult Lgr5⁺ stem cell. Nat. Med. 18, 618–623. 10.1038/nm.2695 22406745

[B305] Yun S. M. Kim S. H. Kim E. H. (2019). The molecular mechanism of transforming growth factor-β signaling for intestinal fibrosis: a mini-review. Front. Pharmacol. 10, 162. 10.3389/fphar.2019.00162 30873033 PMC6400889

[B306] Zaidi D. Bording-Jorgensen M. Huynh H. Q. Carroll M. W. Turcotte J. F. Sergi C. (2016). Increased epithelial gap density in the noninflamed duodenum of children with inflammatory bowel diseases. J. Pediatr. Gastroenterol. Nutr. 63, 644–650. 10.1097/MPG.0000000000001182 26933801

[B307] Zeissig S. Burgel N. Gunzel D. Richter J. Mankertz J. Wahnschaffe U. (2007). Changes in expression and distribution of claudin 2, 5 and 8 lead to discontinuous tight junctions and barrier dysfunction in active Crohn's disease. Gut 56, 61–72. 10.1136/gut.2006.094375 16822808 PMC1856677

[B308] Zhao R. Fallon T. R. Saladi S. V. Pardo-Saganta A. Villoria J. Mou H. (2014). Yap tunes airway epithelial size and architecture by regulating the identity, maintenance, and self-renewal of stem cells. Dev. Cell 30, 151–165. 10.1016/j.devcel.2014.06.004 25043474 PMC4130488

[B309] Zhou J. Alvarez-Elizondo M. B. Botvinick E. George S. C. (2012). Local small airway epithelial injury induces global smooth muscle contraction and airway constriction. J. Appl. Physiol. (1985) 112, 627–637. 10.1152/japplphysiol.00739.2011 22114176 PMC3289432

[B310] Zuo L. Kuo W. T. Cao F. Chanez-Paredes S. D. Zeve D. Mannam P. (2023). Tacrolimus-binding protein FKBP8 directs myosin light chain kinase-dependent barrier regulation and is a potential therapeutic target in Crohn's disease. Gut 72, 870–881. 10.1136/gutjnl-2021-326534 35537812 PMC9977574

